# A high-performance 4 nV (√Hz)^−1^ analog front-end architecture for artefact suppression in local field potential recordings during deep brain stimulation

**DOI:** 10.1088/1741-2552/ab2610

**Published:** 2019-10-09

**Authors:** Konstantinos Petkos, Thomas Guiho, Patrick Degenaar, Andrew Jackson, Peter Brown, Timothy Denison, Emmanuel M Drakakis

**Affiliations:** 1 Department of Bioengineering, Imperial College London, London, United Kingdom; 2 Center for Neurotechnology, Imperial College London, London, United Kingdom; 3 Institute of Neuroscience, Newcastle University, Newcastle upon Tyne, United Kingdom; 4 School of Engineering, Newcastle University, Newcastle upon Tyne, United Kingdom; 5 MRC Brain Network Dynamics Unit, University of Oxford, Oxford, United Kingdom; 6 Nuffield Department of Clinical Neurosciences, University of Oxford, Oxford, United Kingdom

**Keywords:** DBS, artefact suppression, high-performance analog front-end, LFP (/*E* × *G*) bioinstrumentation, analog filtering

## Abstract

**Objective:**

Recording of local field potentials (LFPs) during deep brain stimulation (DBS) is necessary to investigate the instantaneous brain response to stimulation, minimize time delays for closed-loop neurostimulation and maximise the available neural data. To our knowledge, existing recording systems lack the ability to provide artefact-free high-frequency (>100 Hz) LFP recordings during DBS in real time primarily because of the contamination of the neural signals of interest by the stimulation artefacts.

**Approach:**

To solve this problem, we designed and developed a novel, low-noise and versatile analog front-end (AFE) that uses a high-order (8th) analog Chebyshev notch filter to suppress the artefacts originating from the stimulation frequency. After defining the system requirements for concurrent LFP recording and DBS artefact suppression, we assessed the performance of the realised AFE by conducting both *in vitro* and *in vivo* experiments using unipolar and bipolar DBS (monophasic pulses, amplitude ranging from 3 to 6 V peak-to-peak, frequency 140 Hz and pulse width 100 *μ*s). A full performance comparison between the proposed AFE and an identical AFE, equipped with an 8th order analog Bessel notch filter, was also conducted.

**Main results:**

A high-performance, 4 nV ${\left(\sqrt{\text{Hz}}\right)}^{-1}$ AFE that is capable of recording nV-scale signals was designed in accordance with the imposed specifications. Under both *in vitro* and *in vivo* experimental conditions, the proposed AFE provided real-time, low-noise and artefact-free LFP recordings (in the frequency range 0.5–250 Hz) during stimulation. Its sensing and stimulation artefact suppression capabilities outperformed the capabilities of the AFE equipped with the Bessel notch filter.

**Significance:**

The designed AFE can precisely record LFP signals, in and without the presence of either unipolar or bipolar DBS, which renders it as a functional and practical AFE architecture to be utilised in a wide range of applications and environments. This work paves the way for the development of externalized research tools for closed-loop neuromodulation that use low- and higher-frequency LFPs as control signals.

## 1 Introduction

Increasing evidence suggests that local field potential (LFP) oscillations in the beta frequency band (13–30 Hz) can be consistently picked up in the subthalamic nucleus (STN) of patients with Parkinson’s disease (PD) and that their strength correlates with the severity of the disease and the efficacy of therapy (Stanslaski *et al* 2012, Little *et al* 2013). However, the last decade of LFP analysis also focused on spectral power extraction from higher frequency bands, such as high gamma (60–80 Hz) and 300 Hz (270–330 Hz) (Arlotti *et al* 2016). The power of these oscillations also correlates with PD motor symptoms and clinical conditions, thus being eligible as a biomarker (Priori *et al* 2013).

As a result, activity in the aforementioned frequency bands during stimulation could potentially be used to monitor disease progression, assess the effects of therapy and direct patient treatment towards more effective therapeutical strategies. Moreover, maintaining sensing during stimulation, rather than eliminating the available neural data by simply blanking the signal chain during stimulation, might also be vital for closed-loop neurostimulation systems. Simultaneous neural recording and stimulation could help to maximise treatment effectiveness for patients suffering from epilepsy. In an episodic disorder, such as epilepsy, maintaining sensing during stimulation helps minimizing the temporal delay between seizure detection and adaptation of the stimulation to achieve the most effective therapy (Stanslaski *et al* 2012). Finally, observations during stimulation could also reveal novel neural activity patterns that are not present in neural tissue in the absence of stimulation. This could uncover new biomarkers of serious neurological disorders previously masked by stimulation.

However, the large difference between the amplitude of the stimulation pulses and the relevant underlying neural activity leads to the appearance of stimulation artefacts, which impede the accurate recording of neural signals and the processing of potential biomarkers. More specifically, the normal amplitude of LFP signals can range from a few microvolts (e.g. in the basal ganglia) (Goldberg 2004) to hundreds of microvolts in the cortex. Hence, it is clear that the magnitude of LFPs is approximately 100–120 dB (five to six orders of magnitude) smaller than that of the stimulation pulses. Therefore, the design of an AFE that can record weak neural signals (in *μ*V range) in the presence of strong stimulation artefacts (in Volts range) without being saturated, is, perhaps, the most difficult challenge associated with the strategy of concurrent sensing and stimulation.

To alleviate this problem, Rossi *et al* designed an artefact-free recording system for acquisition of LFPs from the DBS lead positioned in the STN (Rossi *et al* 2007). The stimulation artefact at 130 Hz and the higher harmonics were separated from the neural signals of interest in the frequency domain using a 10th order analog low-pass filter at 40 Hz. This high-order filter was formed by cascading five 2nd order Sallen-Key low-pass filters, designed using Butterworth coefficients. The advantages of this system are its high gain of 100 dB and its high common mode rejection ratio (CMRR) of 130 dB. However, that front-end suppresses the stimulation interference by significantly restricting the bandwidth of the recorded LFPs and it requires a ± 15 V supply to operate. Another method to remove stimulation artefacts is post-filtering (Al-ani *et al* 2011). In this case, an artefact-free biomarker is produced by subtracting the template of the stimulation signal from the recorded signal. However, this method degrades the signal quality (Parastarfeizabadi and Kouzani 2017). Furthermore, it may not operate correctly in a closed-loop DBS setting where the stimulation rate may fluctuate (Parastarfeizabadi and Kouzani 2017).

Stanslaski *et al* designed an implantable, chronic, adaptive DBS device that benefits from an LFP/ECoG sensor (Stanslaski *et al* 2012). This device, which was successfully validated in an ovine model of epilepsy by measuring hippocampus seizure activity during and after stimulation, has been chronically implanted in humans (Swann *et al* 2016). A support vector machine (SVM) classification algorithm with spectral fluctuation processing capabilities was used to separate the biomarker from the stimulation artefact. The suggested device fits in a 39 cm^3^ volume, employing front-end band-pass filtering which ensures that the instrumentation amplifier (INA) operates within its normal range. However, an analog third-order low-pass filter at 100 Hz is used to filter chopping clock interference and stimulation interference, thus limiting the available bandwidth for LFP recording. Moreover, the authors found that interactions of stimulation artefact and sampling clock can create an aliased signal in the measurement band.

Finally, Pinnell *et al* introduced a miniature wireless system weighing 8.5 g (including battery) for rodent use that combined multichannel DBS and LFP recordings (Pinnell *et al* 2015). Its performance was verified in a working memory task that involved 4-channel fronto-hippocampal LFP recording and bilateral constant-current fimbria-fornix DBS. The wireless system was capable of simultaneous recording and stimulation for a signal bandwidth between 1.5 and 100 Hz. However, the activation of DBS resulted in prominent stimulation artefacts on the raw LFP trace consisting of both harmonic repetitions of the stimulus frequency, and aliasing artefacts (Pinnell *et al* 2015). The proposed way to alleviate this problem was to introduce relatively lower-intensity stimulation parameters and apply a low-pass filter below 80 Hz on the recorded signals (Pinnell *et al* 2015).

All in all, despite the advances in concurrent neural sensing and stimulation, the issue of stimulation artefact in the recorded LFP signals has not been fully addressed yet in the existing DBS systems (Parastarfeizabadi and Kouzani 2017). This paper focuses on the interface between the neural tissue and the analog front-end (AFE) (which amplifies the neural signals of interest and suppresses stimulation artefacts) prior to digitization and presents the design and testing of a novel AFE architecture, which enables the reliable recording of low- and higher-frequency LFP signals during either unipolar or bipolar DBS.

## 2 Methods

### 2.1 System requirements, design and implementation of the AFE

As already stated in the introduction, the power of the oscillations at physiologically significant bands, such as theta (4–7 Hz), alpha (8–11), low beta (12–20 Hz), high beta (20–35 Hz), high gamma (60–80 Hz), and 300 Hz (270–330 Hz) correlates with PD motor symptoms and clinical conditions (Priori *et al* 2013). Based on these findings, our aim was to design and assess a versatile AFE that provides the passband needed to investigate the existence of possible biomarkers in these frequency bands. Moreover, since the LFP spectral content varies among patients (Arlotti *et al* 2016), the extended passband offered by the proposed AFE could lead to a more in-depth analysis of the spectral content recorded from each patient, facilitating the personalization of treatment for patients suffering from PD.

The proposed AFE is specifically designed to acquire LFPs from DBS electrodes placed in the STN. Post-operative LFPs are usually differentially recorded from two DBS electrode contacts and referred to an electrode placed on the scalp (Rossi *et al* 2007). Differential LFP recording offers the advantage of limiting volume conduction (Gabriel *et al* 2018) and leveraging the CMRR of the front-end amplifier to reduce the artefacts originating from DBS. The requirements for signal acquisition are summarized in table 1. It is clear that a high gain, CMRR and dynamic range along with low noise levels (Zhou *et al* 2018) were required in order to ensure high-performance LFP recording during or without the presence of stimulation. Furthermore, according to the literature (Denison *et al* 2007), when platinum–iridium (PtIr) DBS electrodes are used for recording LFP signals (our case), adequate rejection of differential dc offset voltages that are in the order of tens of millivolts is required. Regarding stimulation artefact suppression, the requirement was to extend the available bandwidth for LFP recording during stimulation beyond the limit of 100 Hz, which is the bandwidth offered by the existing DBS devices for recording neural signals during stimulation.

**Figure 1:**
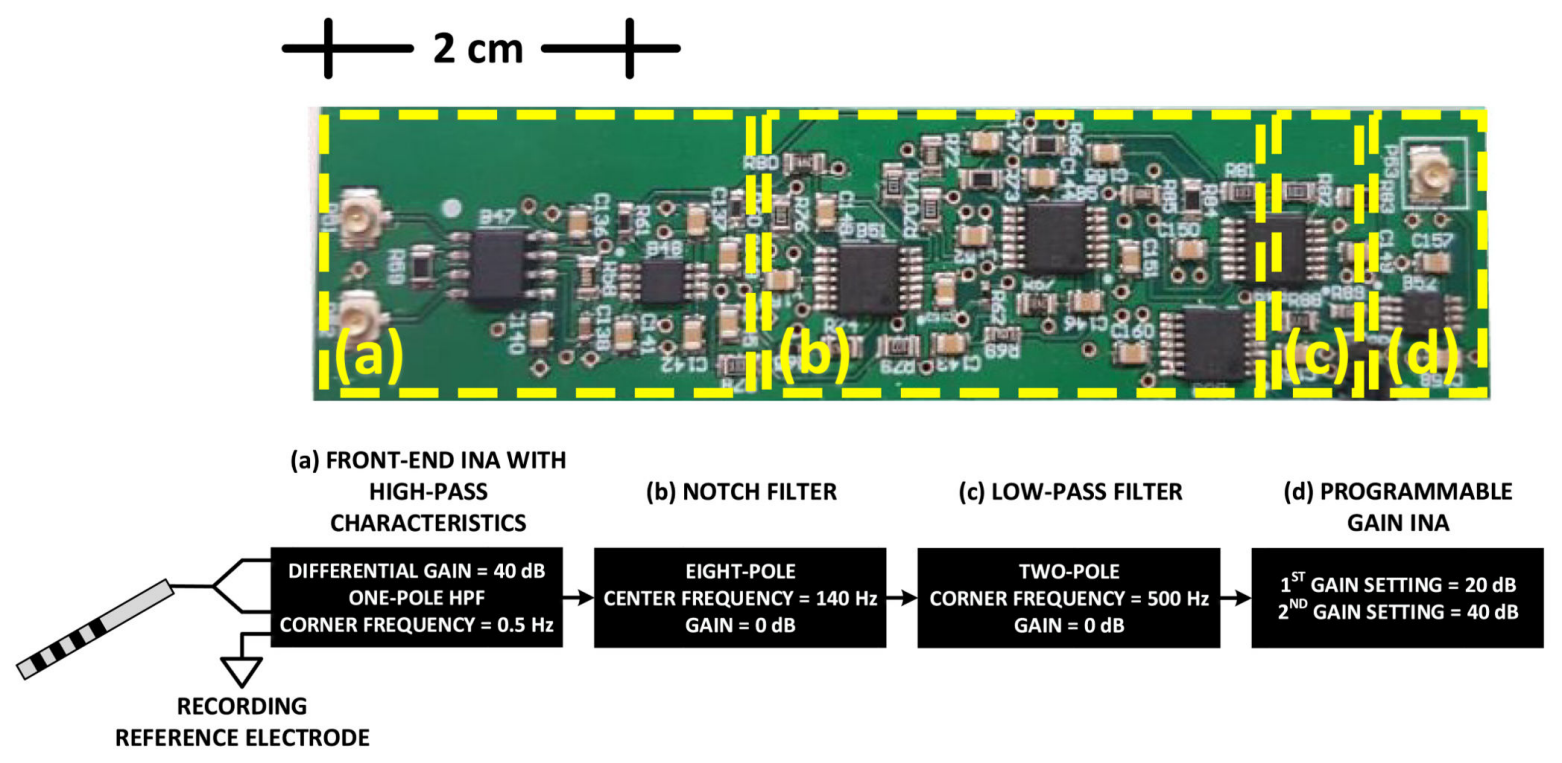
Architecture of AFE design for artefact-free LFP recording during DBS. The AFE consists of (a) a differential pre-amplification stage with high-pass characteristics, which suppresses the CMAV, (b) an 8th order analog notch filter that suppresses the main frequency of the DMAV, (c) a 2nd order analog low-pass filter that suppresses the high-frequency harmonics of the DMAV and (d) a final amplification stage that uses a programmable gain instrumentation amplifier to achieve the required gain. Two AFEs, based on the architecture presented above, have been designed. They only differ in their second stage, where the first AFE (Chebyshev notch channel) employs an 8th order Chebyshev notch filter, whereas the second AFE (Bessel notch channel) employs an 8th order Bessel notch filter.

To meet the requirements for data acquisition, we designed and implemented an AFE consisting of four main stages (figure 1): (i) a differential pre-amplification stage with high-pass characteristics, which suppresses the common mode artefact voltage (CMAV); (ii) an 8th order analog notch filter that suppresses the main frequency of the differential mode artefact voltage (DMAV); (iii) a 2nd order analog low-pass filter that suppresses the high-frequency harmonics of the DMAV and defines the passband of the system; and (iv) a final amplification stage that uses a programmable gain INA to achieve the required gain.

The pre-amplification stage consists of an ultralow noise INA (model AD8429, Analog Devices, USA). Taking into consideration that the high pulse amplitudes of 2–3.5 V in a typical stimulation therapy are up to six orders of magnitude larger than the neural signals of interest, which typically are in the order of 1–10 *μ*V when measured from DBS electrodes (Stanslaski *et al* 2012), an artefact suppression strategy has to be employed. The strategy followed in this design was to initially achieve a significant suppression of the CMAV by exploiting the high CMRR offered by the front-end INA and thus avoiding the use of input protective diodes, or passive high-pass filters that would increase the noise and decrease the CMRR of the AFE (Casas *et al* 2009). Therefore, the gain of the front-end INA was set to 40 dB in order to provide a high CMRR value and thus satisfied the imposed requirement on the CMRR of the system.

**Figure 2:**
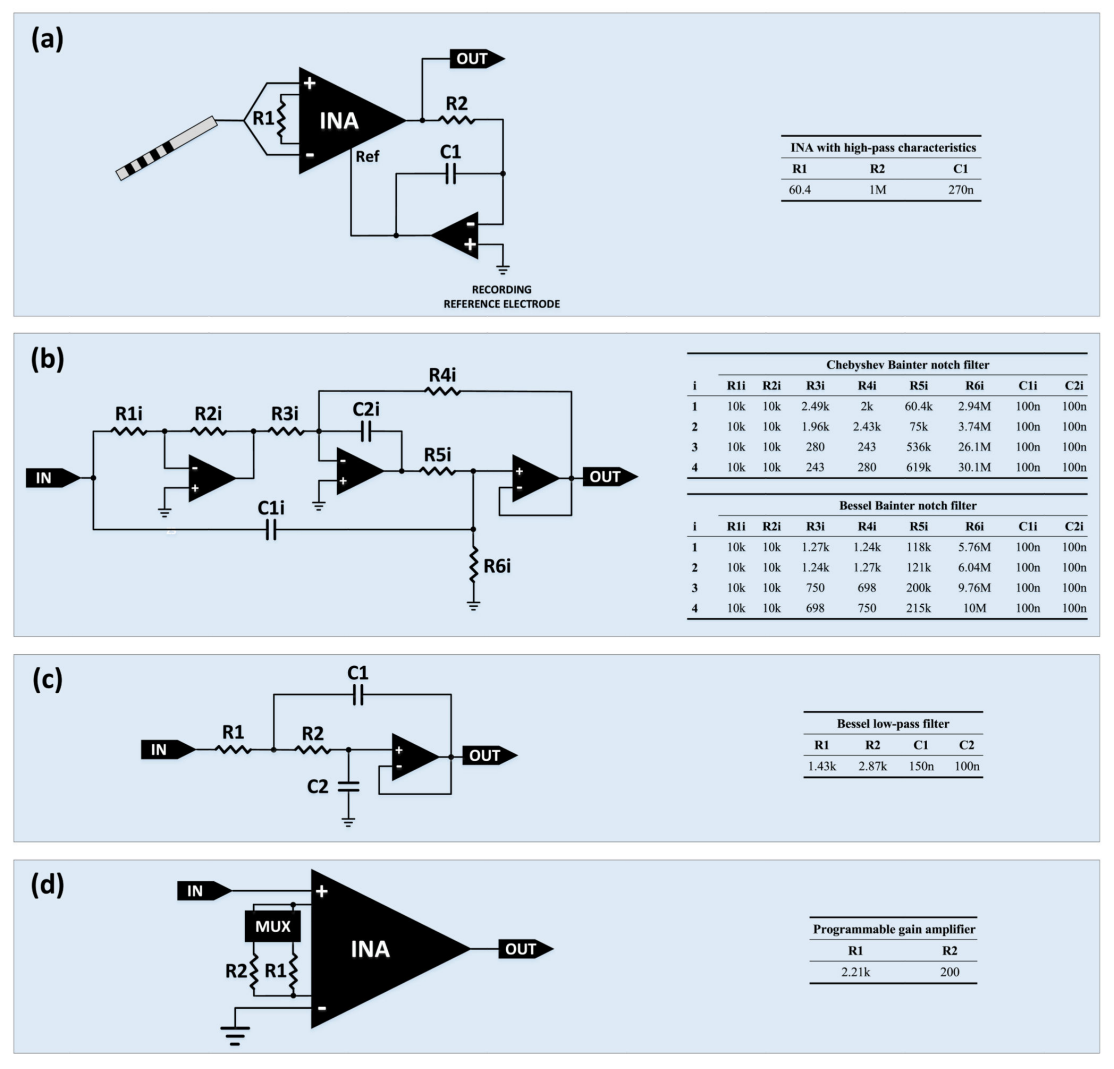
Graphical representation of the four blocks constituting the AFE architecture. The resistors and capacitors included in these blocks are characterized by a tolerance of 0.1% and 10%, respectively. (a) The signals coming from two contacts of the DBS electrode are subtracted and amplified with a gain of 40 dB by an INA with high-pass characteristics (the high-pass knee frequency was set at 0.5 Hz). (b) An eight-pole Bainter 140 Hz notch filter is used to suppress the main frequency of the stimulation artefacts. (c) A two-pole 500 Hz Sallen-Key low-pass filter is used to suppress the high-frequency harmonics of the stimulation artefacts and define the passband of the system. (d) An INA provides either 20 dB or 40 dB amplification, which is digitally determined via a multiplexer.

Another challenge that has to be taken into account in the design of the AFE is that the placement of a metallic electrode in the tissue results in charge redistribution, creating a capacitive double layer that can lead to significant polarization voltages (Merrill *et al* 2005). These offsets can easily saturate the high-gain front-end INA and must be adequately rejected (Spinelli *et al* 2003, Denison *et al* 2007). The strategy followed in this design was to introduce an active feedback integrator (Nikola *et al* 2001) (figure 2(a)), implemented with a single operational amplifier (OPA) (model ADA4522, Analog Devices, USA). The addition of this OPA leads to the formation of the following transfer function:

(1) $$\text{TF}\left(s\right)=\frac{V_{\text{out}}\left(s\right)}{V_{\text{dif}\;\text{f}}\left(s\right)}=\frac{\text{DG}}{1+\left(\text{DG}\times \frac{K}{s}\right)}.$$

Where *V*_out_ is the output voltage of the active feedback integrator, *V*_diff_ is the differential input voltage, DG is the differential gain, *K* is the integrator slope and *s* (=*jω*, where *ω* is the angular frequency in rad/sec) is the complex variable representing frequencies. Clearly, as *ω* decreases, the magnitude of the TF decreases (for *ω* → 0 rad/sec, the TF magnitude approaches 0), whereas when *ω* increases, the magnitude of TF increases (for *ω* → ∞rad/sec, the TF magnitude approximates the value of DG), which is in accordance with the function of a high-pass filter. The zero introduced by the integrator was set at 0.5 Hz.

The analog filtering stages (notch and low-pass) were introduced between the pre-amplification and the final amplification stages in order to suppress the DMAV. More specifically, an eight-pole Bainter notch filter (figure 2(b)) was designed and introduced in the signal chain to suppress the main frequency of the DMAV. Since 140 Hz DBS has proven to improve limb bradykinesia (Blumenfeld *et al* 2017) and continuous high frequency stimulation (130–180 Hz) of subcortical motor nuclei has proven to be highly effective in suppressing PD motor symptoms, and tremor observed in essential and dystonic tremor (Cagnan *et al* 2017), the center frequency of the aforedescribed analog notch filter was chosen to be equal to 140 Hz. Moreover, the stopband of the notch filter was tuned between 125 and 155 Hz. It is important to note here that, according to (Arlotti *et al* 2016), no biomarkers for PD were found in this frequency band though research continues (Shimamoto *et al* 2013, De Hemptinne *et al* 2015).

Two different versions of this notch filter were designed and tested in order to assess which of those two implementations is the most suitable for being placed in the second stage of the final AFE architecture. The first version was a 0.5 dB Chebyshev approximation while the second version was a Bessel approximation. Chebyshev and Bessel approximations were chosen because our aim was to thoroughly investigate the tradeoff between a steep filter roll-off (Chebyshev filters) and an excellent transient response to a step/pulse input thanks to a linear phase response (Bessel filters) (Karki 2002). Both steep roll-off and good transient and phase response are required in neuromodulation. The former is explained by the fact that the proximity of the sensing to the stimulation and the low magnitude of the neural signal relative to the stimulation require the design of filters that can sufficiently suppress the stimulation artefacts, while the latter is required in order to achieve minimally distorted recording of neural signals.

The low-pass filtering stage (figure 2(c)) includes a two-pole classic Sallen-Key low-pass filter designed using Bessel coefficients to ensure an excellent transient response. The role of this filter is to suppress the high-frequency harmonics of the DMAV and define the passband of the system. The objective of this effort was to reliably extract LFP signals during DBS by applying techniques in the analog domain to avoid saturation. As a result, having ensured that the LFP signals will reach the analog-to-digital-converter (ADC), further *a posteriori* low-pass filtering in the digital domain can be applied to completely remove higher harmonics coming from stimulation. Taking into account the aforementioned objective and the fact that a higher order low-pass filter would add extra components that would further increase complexity and possibly power consumption of the AFE and would occupy more space on the final printed circuit board (PCB), a low-order filter was introduced at this stage.

The final amplification stage (figure 2(d)) includes a single-ended amplification with a gain of either 20 dB or 40 dB. An INA (model AD8422, Analog Devices, USA) with its negative input grounded was used to amplify the signals coming from the low-pass filter of the previous stage. The gain is digitally programmable and is determined by a multiplexer (model ADG1404, Analog Devices, USA). The first three stages (differential pre-amplification, notch and low-pass filtering) are supplied with ± 5 V to ensure that an adequate headroom is provided to eliminate the risk of saturation coming from electrode dc offsets and stimulation artefacts. However, the fourth (last) stage is supplied with ± 2.5 V to be able to interface with high-performance and low-power commercial ADC chips (e.g. model ADS1298, Texas Instruments, USA). The fundamental building block for the design of the filtering stages is the OPA ADA4522 by Analog Devices. Finally, the resistors and capacitors included in this four-stage architecture are characterized by a tolerance of 0.1% and 10%, respectively.

The two designed channels (Chebyshev and Bessel notch channel) are powered by a medical DC/DC converter (model THM 10-0521WI by Traco power), which provides a reinforced isolation system for 5000 VACrms isolation and a very low leakage current of less than 2 *μ*A. On the isolated side of the PCB hosting the designed AFEs, a low dropout voltage regulator (model TPS7A7001DDA from Texas Instruments) is used to convert the +5 V originating from the positive (isolated) output of the DC/DC converter into +2.5 V, while a linear voltage regulator (model LM337IMP/NOPB from Texas Instruments) is used to convert the −5 V originating from the negative (isolated) output of the DC/DC converter into −2.5 V.

### 2.2 *In vitro* experimental setup for artefact suppression testing

**Figure 3:**
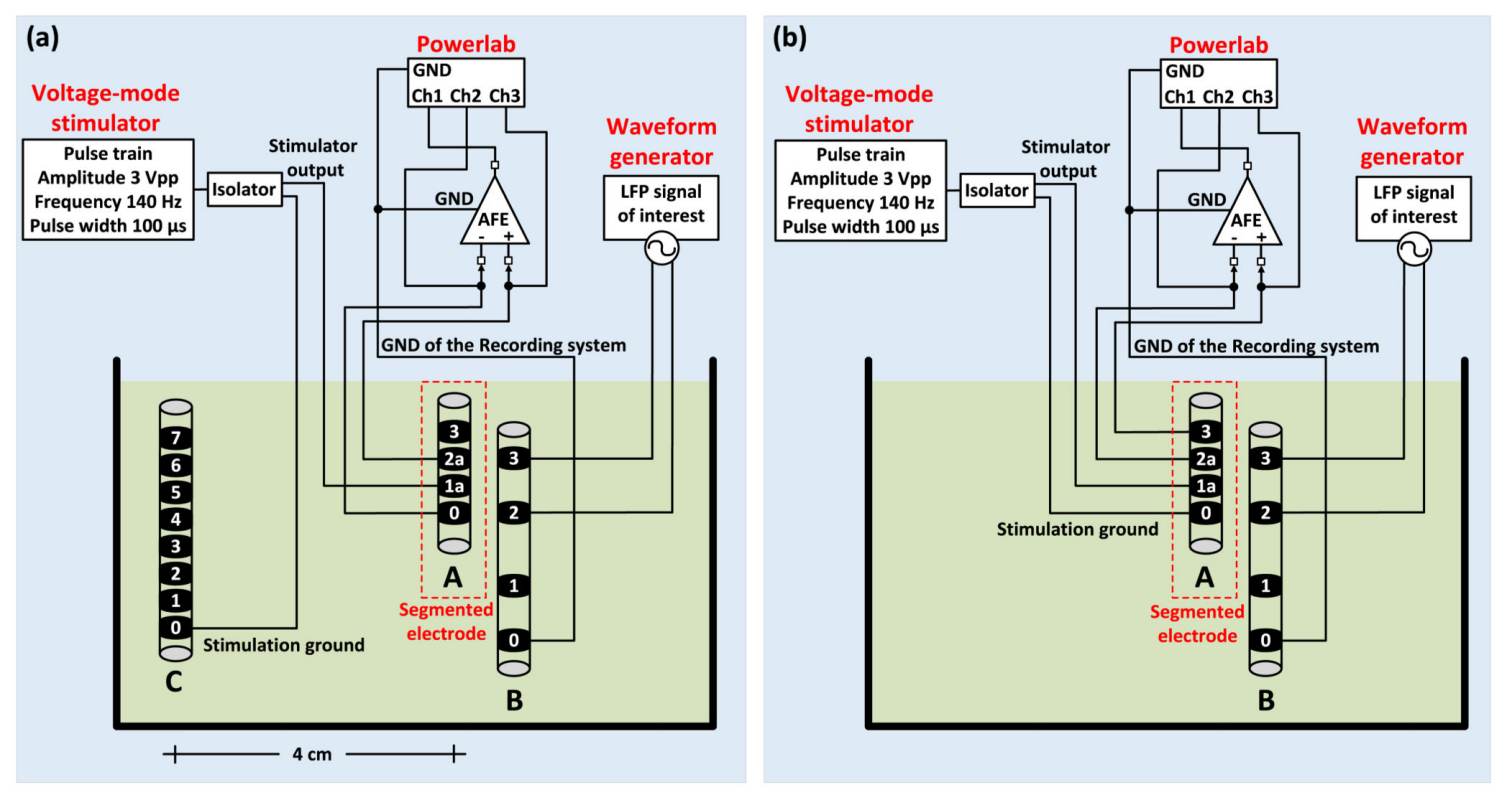
The *in vitro* experimental setup for unipolar (a) and bipolar (b) stimulation. A DBS electrode (electrode A in (a) and (b), model DB-2201, Boston Scientific Neuromodulation) was placed in a glass container filled with tyrode solution at room temperature. The monophasic stimulation pulses (3 V peak-to-peak amplitude, 140 Hz frequency and 100 *μ*s pulse width) were delivered by means of a commercial stimulator (Grass, Astromed, Inc., USA) and the LFP signals (representing the LPF signals recorded from the human neural tissue in a typical post-operative LFP recording session) were injected to the solution by an Agilent 33220A waveform generator. The LFP signals were injected to the solution as a differential signal through a second electrode (electrode B in (a) and (b), model 401261, St. Jude Medical). One of the four contacts of electrode B was connected to the ground of the recording system. In both unipolar and bipolar settings the stimulation ground was electrically isolated from the mains by using a commercial isolator (SIU5 stimulus isolation point, Grass, Astromed, Inc., USA). The LFP signals recorded by the proposed AFE were digitized at a sampling frequency of 20 kSPS (samples per second) and depicted on a computer by the Powerlab data acquisition system (ADInstruments). (a) In the unipolar stimulation setting, we sense differentially and symmetrically in space about the unipolar stimulation contact 1a of electrode A by sensing across the two nearest, equi-distant to contact 1a, neighbour contacts (contacts 0 and 2a). However, since the surface areas of contacts 0 and 2a differ, the sensing is not completely symmetrical and thus some differential-mode interference from stimulation is expected to appear and be suppressed by the analog notch filter of the proposed AFE. The anode (ground) of the stimulator was connected to one of the contacts of a third electrode (electrode C), which is the 8-contact Vercise DBS lead (Boston Scientific). Electrode C was placed approximately 4 cm away from the stimulation site and represents the case of the IPG, which acts as an anode in the unipolar stimulation setting. (b) In the bipolar stimulation setting, two contacts of electrode A (0 and 1a) were used for stimulation (anode and cathode of the stimulator) and another two for recording (2a and 3).

An *in vitro* experimental setup for unipolar (figure 3(a)) and bipolar (figure 3(b)) stimulation was prepared to reproduce the stimulation and recording conditions of a typical post-operative LFP recording session. The DBS electrode used in the experiments (electrode A in figures 3(a) and (b), model DB-2201, Boston Scientific Neuromodulation) is a directional eight-contact segmented DBS lead (Rossi *et al* 2016). We placed the DBS electrode in a glass container filled with tyrode solution (128.2 mM of NaCl, 1.3 mM of CaCl_2_, 4.7 mM of KCl, 1.05 mM of MgCl_2_, 1.19 mM of NaH_2_PO_4_, 20 mM of NaHCO_3_ and 11.1 mM of glucose) at room temperature. The segmented DBS electrode has eight contacts in total, two contacts at the two sides of the electrode (which are contacts 0 and 3 of electrode A in figures 3(a) and (b)) and another six contacts (1a, 1b, 1c, 2a, 2b and 2c).

The monophasic stimulation pulses (3 V peak-to-peak amplitude, 140 Hz frequency and 100 *μ*s pulse width) were delivered by a commercial voltage-mode stimulator (Grass, Astromed, Inc., USA) and the LFP signals (representing the LPF signals recorded from the human neural tissue in a typical post-operative LFP recording session) were injected in the solution by an Agilent 33220A waveform generator. The LFP signals were injected in the solution as a differential signal through a second electrode (electrode B in figures 3(a) and (b), model 401261, St. Jude Medical). One of the four contacts of electrode B was connected to the ground of the recording system. In both unipolar and bipolar settings the stimulation ground was electrically isolated from the mains using a commercial isolator (SIU5 stimulus isolation point, Grass, Astromed, Inc., USA). The output impedance of the SIU5 isolator equals 1 kΩ. The LFP signals recorded by the proposed AFE were digitized and depicted on a computer by means of the Powerlab data acquisition system (Powerlab 16/35, ADInstruments).

In a unipolar configuration one contact on the electrode is set to cathode and the case of the implantable pulse generator (IPG) acts as an anode (Amon and Alesch 2017). In our unipolar stimulation setting (figure 3(a)), we sense differentially and symmetrically in space about the unipolar stimulation contact 1a of electrode A by sensing across the nearest (bilateral to contact 1a) neighbour contacts, i.e. across contacts 0 and 2a. As a result, a significant part of the interference appears as a common-mode signal at the differential sensing pre-amplifier and is rejected by its high CMRR. However, since the surface areas of contacts 0 and 2a differ, the sensing will not be perfectly symmetrical and thus some differential-mode interference caused by the stimulation is expected to appear and be suppressed by the analog notch filter which follows in the AFE’s chain. The anode (ground) of the stimulator was connected to one of the contacts of a third electrode (electrode C), which is the 8-contact Vercise DBS lead (Boston Scientific). Electrode C was placed approximately 4 cm away from the stimulation site and represents the case of the IPG, which acts as an anode in the unipolar stimulation setting.

Finally, in a bipolar configuration one electrode contact is used as the anode and another electrode contact as the cathode, while the case of the IPG is neutral (Schmidt and Van Rienen 2012, Amon and Alesch 2017). In our setup (figure 3(b)), two contacts of electrode A (0 and 1a) were used for stimulation (as the anode and cathode of the stimulator, respectively) and another two for recording (2a and 3). Since contact 2a is closer to the stimulation site in comparison with contact 3, the differential sensing of the contaminating pulses by the front-end INA is asymmetric and thus more differential mode artefacts enter the signal chain. Hence, in the bipolar stimulation setup shown in figure 3(b) the high CMRR of the front-end INA cannot be fully exploited.

## 3 Results

### 3.1 AFE characterization—measured results

As described in section 2.1, two versions of the 8th order Bainter notch filter were designed and introduced in the fundamental AFE architecture shown in figure 1. The aim of this effort was to compare the achieved performance of the two AFE architectures (Chebyshev notch channel versus Bessel notch channel) and decide for the one that is the most suitable for neuromodulation based on the specifications summarized in table 1. In this section, a number of strict tests, which are typically performed on analog electronics to assess their performance in terms of noise, linearity and temporal response are presented and analysed.

#### 3.1.1 Impulse/step response

The impulse function is defined as an infinitely high, infinitely narrow pulse, with an area of unity (Zumbahlen 2008). In practice, when the impulse width is much less than the rise time of the filter, the resulting response of the filter will give a reasonable approximation to the actual impulse response of the filter (Zumbahlen 2008). Rise time is typically defined as the time between 10% response to 90% response of the final value (steady state output) (Ardizzoni 2007). Since the impulse response of the total channel (Chebyshev versus Bessel channel), rather than the sole impulse response of each notch filter, is examined here, the rise time is determined by the front-end first-order high-pass filter and for both channels this is equal to

(2) Risetime=2.2×R×C≈594ms

where *R* = 1 MΩ and *C* = 270 nF (see figure 2(a)).

**Figure 4:**
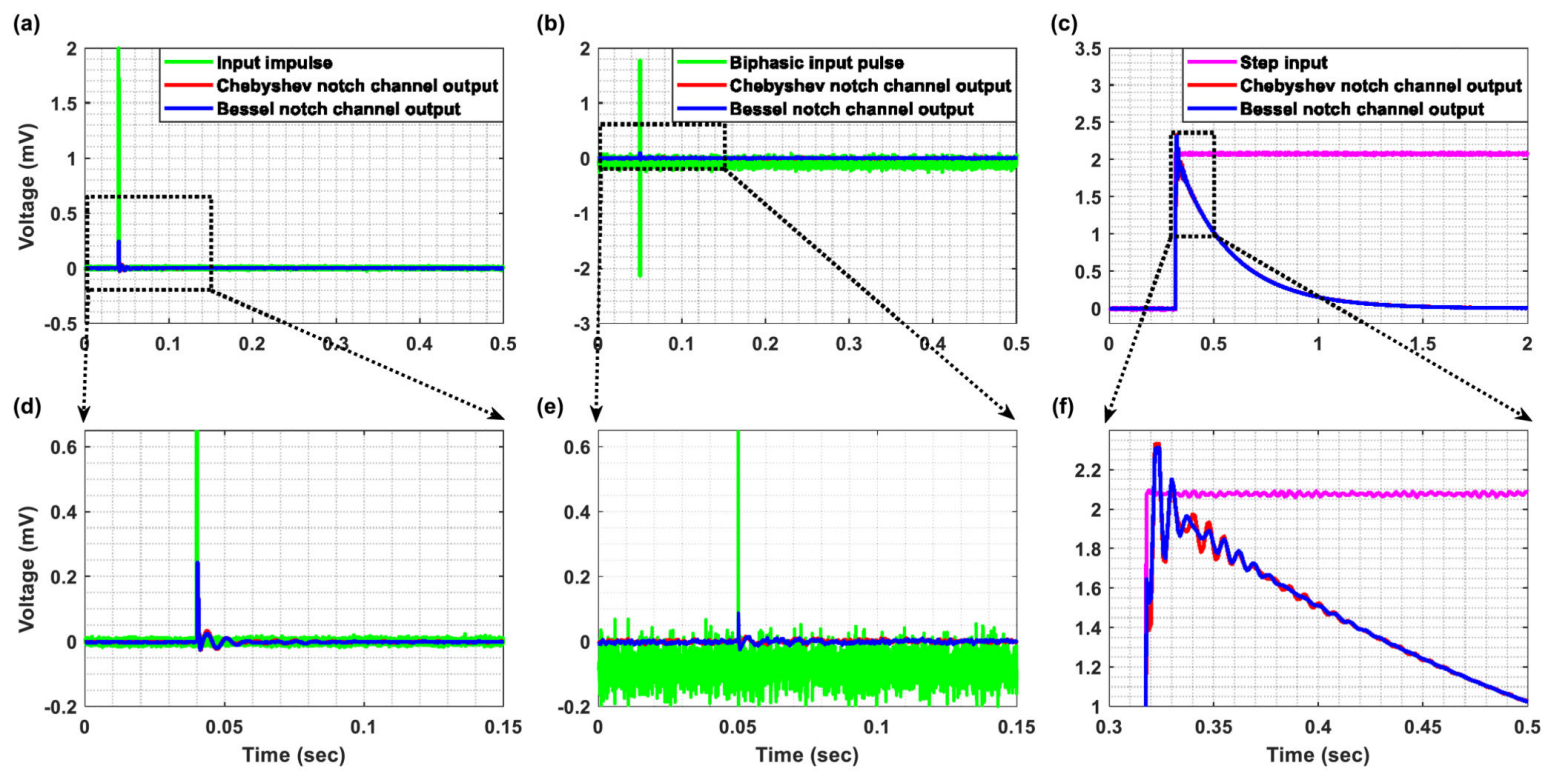
(a) Impulse response, (b) response to a biphasic pulse and (c) step response of the Chebyshev (red line) and Bessel (blue line) notch channels. (d) Chebyshev and Bessel notch channels exhibit approximately the same settling time. (e) The response of both channels to a biphasic pulse exhibits a faster settling in comparison to their corresponding impulse responses. (f) The step response of the Chebyshev notch channel shows a slightly bigger overshoot and ringing in comparison to the Bessel notch channel. As in the case of the impulse response, the differences are not significant.

In figure 4(a), the width of the input impulse was set at 100 *μ*s (which is identical to the DBS pulse duration used in later *in vitro* and *in vivo* experiments) and the amplitude was 2 mV, which is close to the maximum peak amplitude that can be handled by the designed channels (=2.3 mV). Chebyshev and Bessel notch channels exhibit approximately the same settling time (figure 4(d)). Another important test for evaluating the temporal response of the designed AFEs is to supply them with a biphasic input pulse. The responses of the Chebyshev and Bessel notch channels to a biphasic input pulse are shown in figure 4(b). The input pulse was approximately equal to 2 mV for 100 *μ*s and −2 mV for another 100 *μ*s. As anticipated, the responses of both channels to a biphasic input pulse (figure 4(e)) exhibit a faster settling in comparison to their impulse responses (figure 4(d)).

The step response of a filter, which is the integral of the impulse response, is useful in determining the envelope distortion of a modulated signal (Zumbahlen 2008). The two most important features of a filter’s step response are the overshoot and ringing. Overshoot should be minimal for good pulse response and ringing should decay as fast as possible, so as not to interfere with subsequent pulses. Transient response curves cannot provide a completely accurate estimation of the output since, in practice, signals typically are not made up of impulse pulses or steps (Zumbahlen 2008). However, these curves constitute a convenient figure of merit so that transient responses of various filter types can be compared on an equal footing (Zumbahlen 2008). The step response of the Chebyshev notch channel shows a slightly bigger overshoot and ringing in comparison to the Bessel notch channel (figure 4(f)). As in the case of the impulse response, the differences, which are in accordance with the nature of the two notch filters, are not significant. Finally, figure 4(c) reveals the accoupling characteristics of the designed channels. Although the input voltage remains at 2.1 mV, the output voltage returns back to 0 V after a settling time (time needed for the response to reach and stay within 2% of its final value) of:

(3) Settlingtime=4×R×C≈1s.

#### 3.1.2 Bode magnitude plot/noise

**Figure 5:**
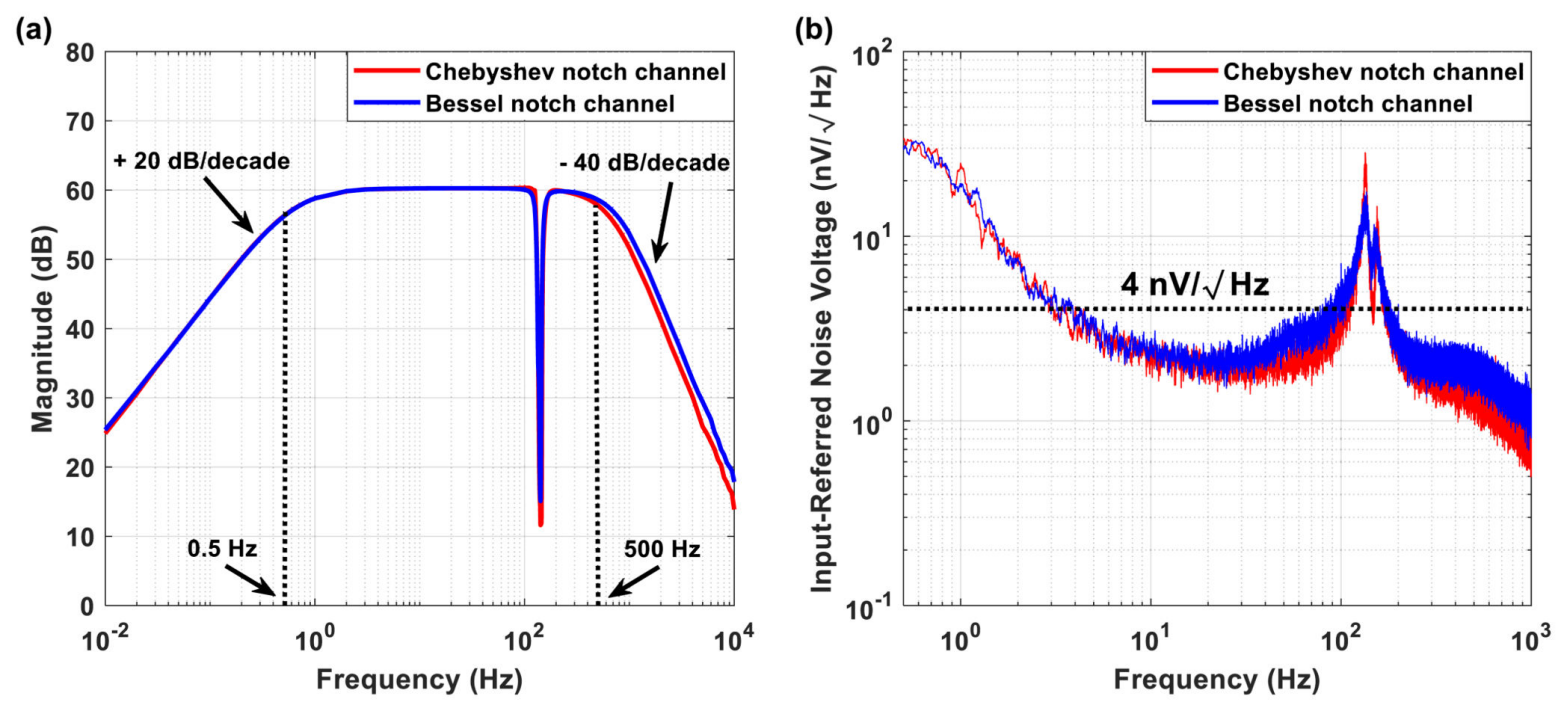
Measured Bode magnitude plot with the gain of both channels set at 60 dB (a) and input-referred noise (b) of the Chebyshev notch channel (red line) and the Bessel notch channel (blue line). (a) Both channels provide a passband between 0.5 and 500 Hz. The roll-off of the high- and the low-pass filters equals +20 dB/decade and −40 dB/decade, respectively, for both topologies. However, the Chebyshev notch channel provides a sharper transition between the passband and the stopband and stronger attenuation at the central frequency of the notch (=140 Hz), compared to the Bessel notch channel. Besides, the Chebyshev notch channel exhibits a flat magnitude response in the passband and approximates the magnitude response of the Bessel notch channel. (b) Based on the input-referred noise graph, it is concluded that both channels are low-noise with the Chebyshev notch channel presenting a slightly better noise performance. Noise power spectral density estimates in the passband for the Chebyshev and the Bessel notch channels are 4 nV ${\left(\sqrt{\text{Hz}}\right)}^{-1}$ and 4.4 nV ${\left(\sqrt{\text{Hz}}\right)}^{-1},$ respectively, with the residual 1/*f* corner estimated at roughly 10 Hz for both channels.

From the Bode magnitude plot shown in figure 5(a), it is clear that both channels provide a passband between 0.5 and 500 Hz and achieve the desired gain of 60 dB. The roll-off of the high- and the low-pass filters equals + 20 dB/decade and −40 dB/decade, respectively, for both topologies. However, the Chebyshev notch channel achieves a sharper transition between the passband and the stopband at 140 Hz, compared to the transition of the Bessel notch channel. Moreover, the Chebyshev notch channel provides stronger attenuation at the central frequency of the notch, which is equal to 140 Hz, compared to the Bessel notch channel. Although the most serious drawback of the Chebyshev approximation is that it allows ripple in the frequency response in order to achieve a faster roll-off (Karki 2002), the proposed Chebyshev notch channel exhibits a flat magnitude response in the passband and approximates the magnitude response of the Bessel notch channel. This is attributed to the fact that it was designed as a 0.5 dB Chebyshev filter and thus the amount of passband ripple is limited.

An input-referred noise voltage graph presents the input noise voltage of a system versus frequency. It is widely used to evaluate the flicker (or 1/*f*) and the thermal noise of a system, as well as the noise corner frequency, which is the point in the frequency spectrum where 1/*f* noise and thermal (or white) noise are equal (Wu *et al* 2013). The input-referred noise was measured by connecting both inputs of the front-end INA to the ground of the PCB, recording the output voltage of the channel and then dividing it by the gain, which was equal to 60 dB. Since there is no passive filtering network before the front-end AD8429 INA chip and the gain of the first stage is sufficiently high (equal to 40 dB), which allows the effective noise factor to be the noise factor of the first stage without an impact on the subsequent stages (Northrop 2012, Poshala *et al* 2014), the input-referred noise of the designed AFEs should approximate the measured input-referred noise reported in the datasheet of the AD8429 chip. The integrated noise of the Chebyshev and Bessel notch channels over the frequency range 0.5–500 Hz was measured and found to be equal to 96 nV rms and 121 nV rms, respectively. Figure 5(b) shows that both channels are low-noise with the Chebyshev notch channel characterised by a slightly better noise performance. Noise power spectral density estimates in the passband for the Chebyshev and the Bessel notch channels are 4 nV ${\left(\sqrt{\text{Hz}}\right)}^{-1}$ and 4.4 nV ${\left(\sqrt{\text{Hz}}\right)}^{-1},$ respectively, with the residual 1/*f* corner estimated at roughly 10 Hz for both channels. Indeed, these measured results are in agreement with the noise measurements reported in the datasheet of the front-end AD8429 INA chip.

#### 3.1.3 Measured results versus specifications

Taking into consideration the previously presented Bode amplitude plot and noise performance of the two channels, the recording capabilities of the Chebyshev notch channel satisfy all of the requirements shown in table 1. Regarding the Bessel notch channel, it satisfies all of the requirements except for the one related to the integrated noise of the channel. The integrated noise of the Bessel notch channel over the frequency range 0.5–500 Hz was measured and found to be equal to 121 nV rms which is higher than the imposed limit of 100 nV rms (table 1). Since the recording capabilities of the Bessel notch channel have not satisfied all of the imposed specifications, measured results only from the Chebyshev notch channel are presented in sections 3.1.4, 3.1.5, 3.2–3.4.

#### 3.1.4 Total harmonic distortion (THD) and intermodulation distortion (IMD)

Due to nonlinearities of electronic components, distortion is generated. Two of the most common ways to assess the linearity of an amplifying system is to specify its THD and its IMD levels.

**Figure 6:**
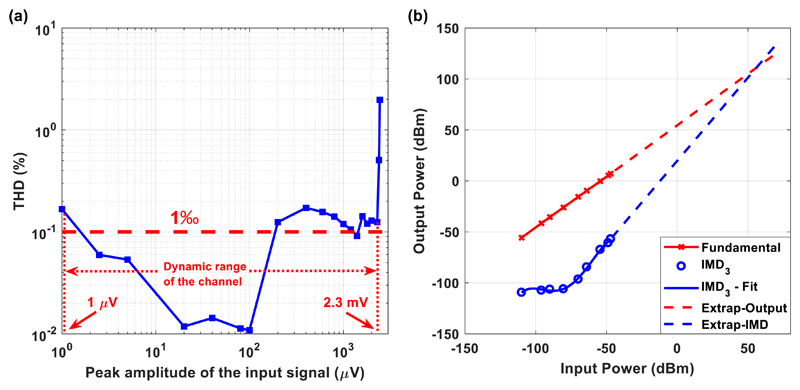
THD (a) and third order IMD (the reference impedance equals 50 Ω) (b) measured with the gain of the Chebyshev notch channel set at 60 dB. (a) After examining the available dynamic range of the channel (from 1 *μ*V peak to 2.3 mV peak), it is clear that the achieved THD is less than 0.2%. (b) The two tones applied to the Chebyshev notch channel were *f*_1_ = 4.9 Hz and *f*_2_ = 5.1 Hz. The output power of a single fundamental tone (in dBm—red line in the graph) and the relative amplitude of the third order IMD_3_ products referenced to a single tone (blue circles in figure (b)) are plotted as a function of the applied input power. The third order intercept line (dashed blue line) is extended to intersect the extension of the fundamental output signal line (dashed red line). This intersection is termed the third order intercept point IP_3_. The calculated IP_3_ is characterized by a relatively high value, which is a positive result since the higher the IP_3_ values the better the linearity of the amplifier and the weaker the output intermodulation products that will be generated at the amplifier’s output.

THD is the ratio of the root-sum-square value of all the harmonics (2×, 3×, 4×, etc) to the rms signal level (Lai 2009). Generally speaking, only the first five or six harmonics are significant in the THD measurement (Lai 2009). In other words, THD measures the nonlinearity of a system, while applying a single sinusoidal signal as its input. The THD of the Chebyshev notch channel for a gain of 60 dB is shown in figure 6(a). After examining the available dynamic range of the channel (from 1 *μ*V peak to 2.3 mV peak), it is clear that the achieved THD is less than 0.2%. Only input sinusoidal voltages with peak amplitudes approaching the highest input voltage (=2.5 mV peak) that can be handled by the rail-to-rail output INA located at the last stage of the AFE present higher THD values.

In general, when a spectrally pure sinusoidal signal passes through an amplifier, various harmonic distortion products are produced depending on the nature and the severity of the non-linearity (Lai 2009). However, simply measuring harmonic distortion levels produced by single tone sinusoidal signals of various frequencies does not convey all the information required to assess the amplifier’s potential performance in a clinical setting, where reliable recording of neural signals is required. Hence, it is often required that an amplifier be evaluated in terms of the IMD product levels produced by two or more specified tones applied at the input of the amplifier (Lai 2009).

Thus, our aim was to not only examine the THD of the Chebyshev notch channel, which is produced by a single tone sinusoidal input, but also to investigate the distortion products produced by two input tones. When two tones of frequencies, *f*_1_ and *f*_2_, are applied to the input of a nonlinear system, they produce second and third order products. The second order products are located at frequencies *f*_2_ + *f*_1_ and *f*_2_ − *f*_1_. The third order products located at frequencies 2*f*_1_ + *f*_2_ and 2*f*_2_ + *f*_1_ can often be filtered out. However, the third order products located at 2*f*_1_ − *f*_2_ and 2*f*_2_ - *f*_1_ are situated close to the main tones *f*_1_ and *f*_2_ and thus it is difficult to be rejected by filtering (Lai 2009). It can be shown that second order IMD levels increase by 2 dB for every 1 dB of input signal increase while the third order IMD amplitudes increase by 3 dB for every 1 dB of input signal increase (Lai 2009).

Third order IMD performance is often specified in terms of the third order intercept point (IP_3_). Two spectrally pure tones are applied to the system. The two tones applied to the Chebyshev notch channel were *f*_1_ = 4.9 Hz and *f*_2_ = 5.1 Hz. In figure 6(b), the output power of a single fundamental tone (in dBm—red line in the graph) and the power of the third order products (blue circles in figure 6(b), defined as IMD_3_) are plotted as a function of input power. It is clear that the fundamental line is characterized by a slope that is equal to 1.

The third order intercept line (dashed blue line) is extended to intersect the extension of the fundamental output signal line (dashed red line). This intersection is termed the third order intercept point (IP_3_) and is a figure of merit for comparing amplifiers’ linearity. The higher the IP_3_ values the more linear the amplifier and the weaker the distortion products at its output. As shown in figure 6(b), the IP_3_ of the Chebyshev notch AFE is characterized by a high value. It should be stressed that this high IP_3_ value of the proposed AFE is a very desirable feature: the non-linearity of the AFE should indeed be very low to avoid artefact coupling into the physiological measurements through intermodulation.

#### 3.1.5 Key properties of the Chebyshev notch AFE

Taking into account all the measured results acquired from the Chebyshev notch channel, the key properties of this channel are summarized in table 2.

### 3.2 Performance evaluation of the AFE based on comparisons with commercial biopotential acquisition devices

To compare our system with available devices, we introduced identical, extremely weak single tones to the Chebyshev notch channel and to a state-of-the-art, commercial biological amplifier (Bioamplifier included in Powerlab 26T, ADInstruments), which is optimized for measuring a wide variety of biological signals such as ECG, EMG and EEG. In the first case, the signals were recorded by the AFE of the Chebyshev notch channel and were digitized by the 16-bit ADC of the Powerlab 16/35 system at 1 kSPS, whereas in the second case the signals were recorded and digitized by the Powerlab 26T (bioamplifier and 16-bit ADC) system at 1 kSPS.

More specifically, a weak sinusoidal single tone (100 nV peak, 25 Hz) was presented to the inputs of the two systems. This weak sinusoidal single tone was provided by the Agilent 33220A waveform generator. However, since the weakest signal that can be injected by the specific generator is a 10 mV peak sinewave, we made use of ohmic attenuators (Cinch Connectivity Solutions) that provided 100 dB attenuation to the signals injected by the waveform generator. A digital low-pass filter at 30 Hz was applied on the recordings of both systems in order to ensure that the noise coming from the front-ends of both systems (integrated noise) stays at levels lower than 100 nV peak (so that the 100 nV peak signal dominates the noise), and be able to compare them on an equal footing. Moreover, the mean values of the signals recorded by the two systems were removed in order to facilitate a more direct comparison between the two AFEs in terms of signal quality.

Regarding the first system (Chebyshev notch channel), its gain was set at 80 dB (or 10 000 V/V) and the range of the Powerlab ADC at ± 10 V (maximum available). The reason behind the choice of applying a gain of 80 dB lies with the fact that a gain of 60 dB would not allow for the amplified signals to overcome the smallest input increment the specific **ADC** can resolve (20/65 536 = 305 *μ*V). Regarding the second system (Powerlab 26T bioamplifier), its recording range was set at ± 100 *μ*V (lowest available), which means that a gain of 100 dB (or 100 000 V/V) was applied upon the input signals by the bioamplifier’s AFE.

**Figure 7:**
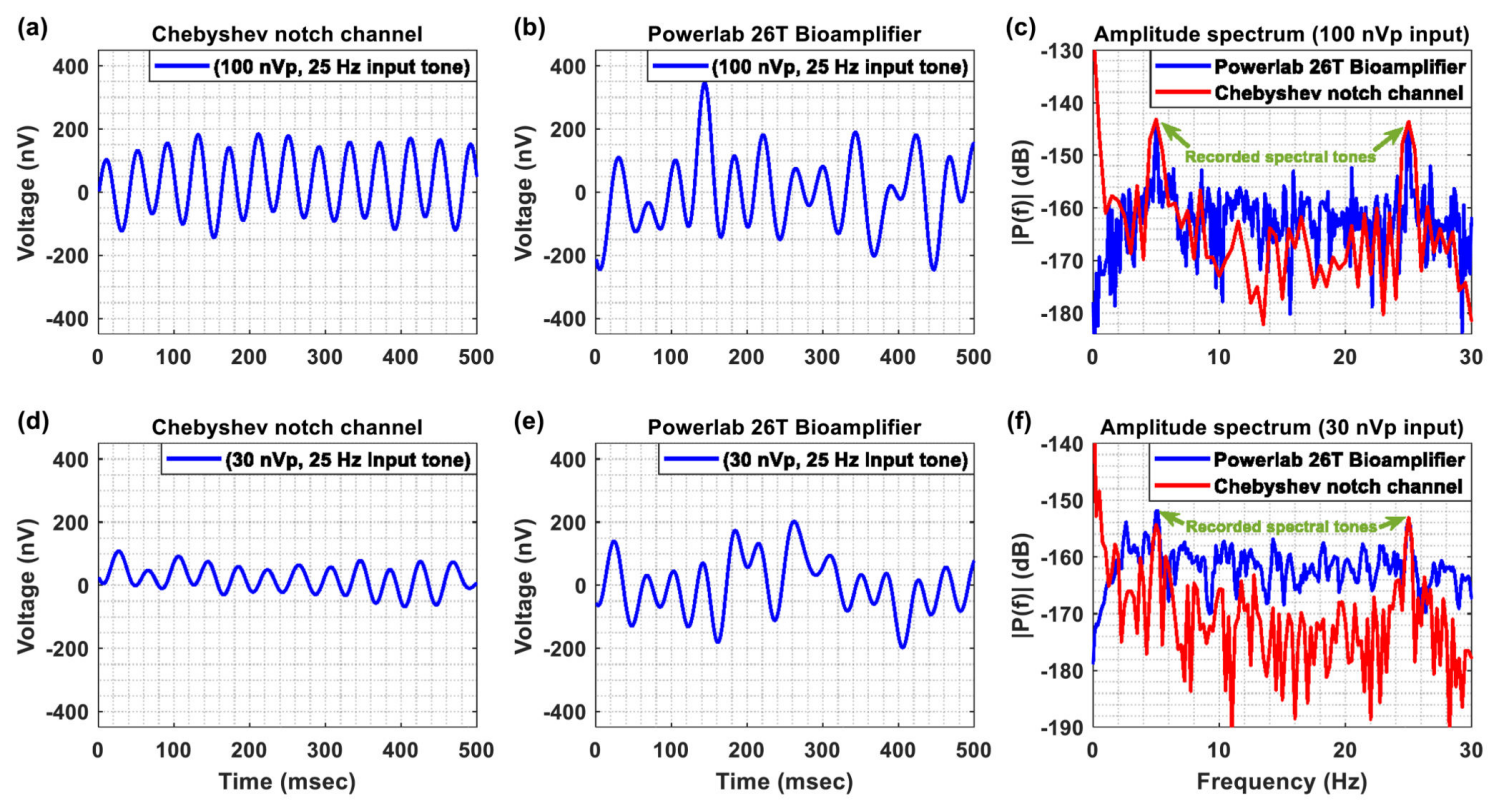
(a) Output voltage (after removing the gain of 80 dB) recorded from the Chebyshev notch channel when a sinusoidal single tone (25 Hz, amplitude 100 nV peak) was injected to the input of the channel. (b) Output voltage recorded from the Powerlab 26T bioamplifier when a sinusoidal single tone (25 Hz, amplitude 100 nV peak) was injected to the input of the system. (c) Amplitude spectrum calculated when two sinusoidal tones, one low-frequency (=5 Hz) and one higher-frequency (=25 Hz) are sequentially injected to the inputs of the two AFEs. The amplitude spectrums of both systems present two spectral peaks at 5 and 25 Hz, which are characterized by the same amplitude. (d) Output voltage (after removing the gain of 80 dB) recorded from the Chebyshev notch channel when a sinusoidal single tone (25 Hz, amplitude 30 nV peak) was injected to the input of the channel. (e) Output voltage recorded from the Powerlab 26T bioamplifier when a sinusoidal single tone (25 Hz, amplitude 30 nV peak) was injected to the input of the system. (f) Amplitude spectrum calculated when two sinusoidal tones, one low-frequency (=5 Hz) and one higher-frequency (=25 Hz), are sequentially injected to the inputs of the two AFEs. The amplitude spectrums of both systems present two spectral peaks at 5 and 25 Hz, which are characterized by the same amplitude.

Figure 7 shows that the Chebyshev notch channel (figure 7(a)) is less vulnerable to DC offsets that exist in the weak sinusoidal signal and can thus provide more stable signal recordings compared to the commercial bioamplifier (figure 7(b)). Next, a second sinusoidal single tone with the same amplitude but lower frequency (=5 Hz) was injected to the inputs of the two AFEs and the amplitude spectrum of the overall recorded signal was calculated (figure 7(c)). It is clear that the spectrums of both systems include visible spectral peaks at the two test frequencies (5 and 25 Hz). Based on the graph, the amplitude (the reference voltage equals 1 V) of each of these two spectral peaks is approximately equal to −143 dB, which is in accordance to the expected theoretical value of

(4) $$\begin{array}{l} \text{Amplitude}=20\times {\text{log}}_{10}\left(\frac{100\times 10^{-9}}{\sqrt{2}}\right)\\ \;\approx -143\;\text{dB}\;\left\{\text{reference}\;\text{voltage}=1\;\text{V}\right\}. \end{array}$$

To push the limits of the Chebyshev notch channel’s recording capabilities towards the noise floor of the system, an extremely weak sinusoidal single tone (30 nV peak, 25 Hz) was presented to the inputs of the two systems. Again, this sinusoidal single tone was provided by the Agilent 33220A waveform generator in combination with attenuators that provided 110 dB attenuation to the signals injected by the waveform generator. A digital low-pass filter at 30 Hz was applied on the recordings of both systems. The gain and digitization settings were left the same with the ones used in the experiment where 100 nV peak test tones were applied.

As anticipated based on the results acquired by the injection of the 100 nV peak test tone, the Chebyshev notch channel (figure 7(d)) provides more stable signal recordings compared to the commercial bioamplifier (figure 7(e)). Next, a second sinusoidal single tone with the same amplitude but lower frequency (=5 Hz) was injected to the inputs of the two AFEs and the amplitude spectrum of the overall recorded signal was calculated (figure 7(f)). It is clear that the spectrums of both systems include visible spectral peaks at the two test frequencies (5 and 25 Hz). It is important to note here that the noise floor of the Chebyshev notch channel (spectrum in red) is lower than the noise floor of the biological amplifier (spectrum in blue). Based on the graph, the amplitude (the reference voltage equals 1 V) of each of these two spectral peaks is approximately equal to −153 dB, which is in accordance to the expected theoretical value of

(5) $$\begin{array}{l} \text{Amplitude}=20\times {\text{log}}_{10}\left(\frac{30\times 10^{-9}}{\sqrt{2}}\right)\\ \;\approx -153\;\text{dB}\left\{\text{reference}\;\text{voltage}=1\;\text{V}\right\}. \end{array}$$

Finally, a 50 s segment of LFP signal recorded (low-pass filtered by a high-order digital low-pass filter at 553 Hz) from the subthalamic nucleus in a patient with PD withdrawn from levodopa was injected by means of a waveform generator to the input of the Chebyshev notch channel. Moreover, in order to ensure that no phase distortion or ringing oscillations are introduced by the analog Chebyshev notch filter when LFP recordings are obtained, the same LFP signal was injected to a commercial high-performance differential amplifier that does not include any analog notch filtering stage in its front-end electronics. The commercial amplifier used in this series of experiments is the DP-301 model (ADInstruments), which has been designed for amplifying weak signals such as extracellular action potentials, and weaker EEG and ECG signals.

However, since the waveform generator is not able to inject signals that are weaker that 10 mV peak, the injected LFP signal at the generator’s output (which was in mV range) had to be attenuated before entering the input of the Chebyshev notch channel. More specifically, four attenuators (Cinch Connectivity Solutions) that provided 80 dB attenuation were used in order to bring the amplitude of the LFP signal injected by the waveform generator down to the level that characterizes the original LPF signal, which is approximately equal to 0.32 *μ*V rms. The spectrum of this signal contains a peak in the beta frequency band (13–30 Hz) and another peak at 80 Hz. The gain of the DP-301 amplifier was set at 80 dB (maximum available) in order to ensure that this instrument will provide a reliable recording of the weak LFP signal. The gain of the Chebyshev notch channel was also set at 80 dB to compare the two systems on an equal footing. Finally, the analog outputs of the two systems were sampled by the ADC of the Powerlab 16/35 system at 1 kSPS (the range was set at ± 2 V so the smallest resolvable input increment of the ADC and the smallest detectable signal by the two systems (Chebyshev notch channel and commercial amplifier) were equal to 61 *μ*V and 6.1 nV, respectively). The analog high-pass filter included in the DP-301 amplifier (cut-off frequency at 1 Hz) was activated so that its temporal response can be compared with the temporal response of the Chebyshev notch channel’s ac-coupled AFE (cut-off frequency at 0.5 Hz) on an equal footing.

**Figure 8:**
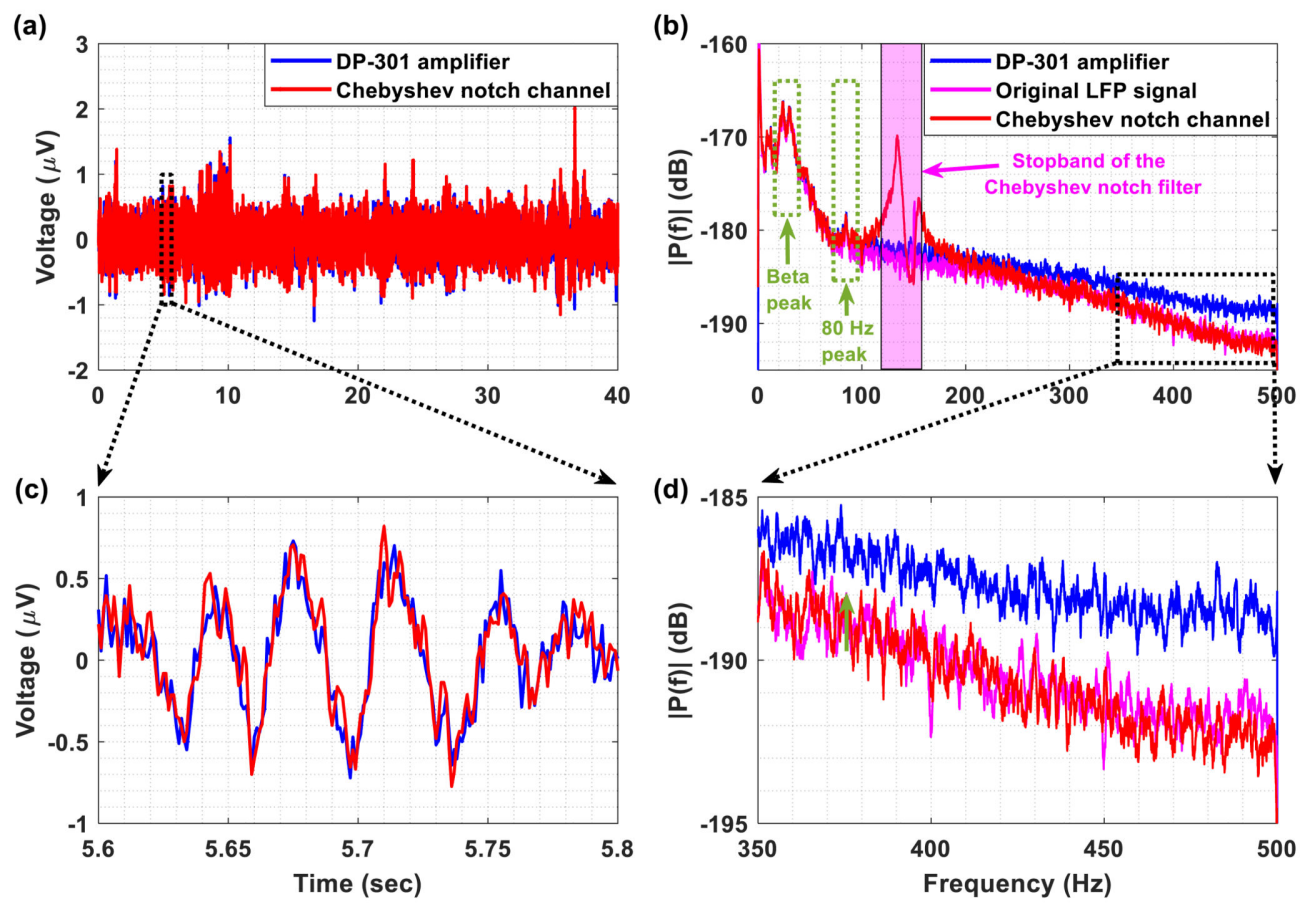
A weak LFP signal is injected into the inputs of the Chebyshev notch channel and the DP-301 commercial differential amplifier (ADInstruments). (a) Comparison of the Chebyshev notch channel’s output (red line—after removing the gain of 80 dB) with the DP-301 amplifier’s output (blue line—after removing the gain of 80 dB) in the time domain. (b) The amplitude spectrum of the Chebyshev notch channel’s output (red line) approximates the amplitude spectrum of the original LFP signal (pink line). (c) The LFP recordings acquired by the Chebyshev notch channel and the DP-301 amplifier approximate each other. This shows that the proposed AFE architecture is capable of recording weak LFP signals without introducing any phase distortion or ringing oscillations. (d) The proposed AFE architecture provides more accurate recording of the high frequencies (*f* > 350 Hz) included in the original LFP signal in comparison to the DP-301 amplifier.

Figure 8(a) depicts the LFP signal recorded by the high-order Chebyshev notch channel (red line) and the DP-301 amplifier (blue line). The two signals approximate each other which shows that the Chebyshev notch channel is able to record the LFP signal without introducing any phase distortion or ringing oscillations (figure 8(c)). Moreover, since the amplitude spectrum of the LFP signal recorded by our channel (figure 8(b)) contains both the beta peak and the peak at 80 Hz, it can be concluded that the proposed AFE architecture can record, save for the stopband frequencies, both the low and high frequencies of the original LFP signal. It is important to note here that the peak (red line) existing in the stopband (125–155 Hz) is introduced by the notch operation (bear in mind figure 5(b)). As is shown in figure 8(b), the noise added by the Chebyshev notch filter does not significantly affect the frequencies below and above the stopband of this filter. However, physiological information should not be sought after in the stopband of the notch (pink region in figure 8(b)).

On the other hand, as is shown in figure 8(a), the stopband noise does not seem to significantly affect the time-domain recording of the Chebyshev notch channel. The normalised root mean square error, or RMSE, between the time-domain LFP signals recorded by the two systems (Chebyshev notch channel and DP-301 amplifier) was measured and found to be equal to 4.6%. This error, which can be considered tolerable taking into account the extremely low amplitude of the specific LFP signal, can be attributed to (1) the fact that the DP-301 amplifier cannot accurately record frequencies of the LFP signal that are higher than 350 Hz (figure 8(d)), (2) the fact that small DC offsets existing in the extremely weak LFP signal are not completely rejected by the two systems, and (3) the noise in the stopband coming from the Chebyshev notch operation.

### 3.3 Evaluation of the artefact suppression capabilities of the AFE by *in vitro* DBS tests

To examine the capability of the proposed Chebyshev notch channel to suppress stimulation artefacts and thus allow artefact-free LFP recording during stimulation, we prepared two *in vitro* setups, one for testing unipolar DBS and one for testing bipolar DBS. The details of these two setups have been given in figure 3 (section 2.2). More specifically, our aim was to investigate whether or not the proposed Chebyshev notch channel could extend the available bandwidth of LFP recording during stimulation, and to further compare its performance with the Bessel notch channel’s performance but with the focus to be on their stimulation suppression capabilities rather than their recording capabilities, which have already been tested (sections 3.1.1 and 3.1.2).

The strategy followed for the tests was to gradually increase the available bandwidth and thus allow more artefacts to affect the recorded signals. At each bandwidth setting, the quality of the recorded signals was assessed. The shortening of the available bandwidth was achieved by the application of a real-time and high-order digital low-pass filter. The first step towards increasing the available bandwidth for recording during stimulation setups, was to define a passband between 0.5 and 140 Hz, with 140 Hz being the stimulation frequency and the central frequency of the notch filters. The next step was to define a passband between 0.5 and 250 Hz to examine the impact of the artefacts coming from the stimulation harmonic at 280 Hz on the recorded signals.

**Figure 9:**
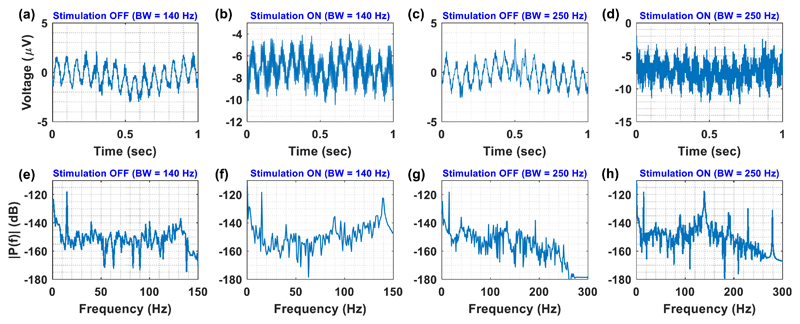
Time and frequency responses of the Chebyshev notch channel, in and without the presence of bipolar stimulation (140 Hz, 3 V peak, 100 *μ*s). The test signal was a sinusoidal single tone with an amplitude of approximately 1 *μ*V peak and a frequency of 15 Hz. (a) Time-domain recording without the presence of stimulation for a passband set from 0.5 to 140 Hz. (b) Time-domain recording in the presence of stimulation for a passband set from 0.5 to 140 Hz. (c) Time-domain recording without the presence of stimulation for a passband ranging from 0.5 to 250 Hz. (d) Time-domain recording in the presence of stimulation for a passband ranging from 0.5 to 250 Hz. (e) Amplitude spectrum of the signals presented in figure (a). (f) Amplitude spectrum of the signals presented in figure (b). (g) Amplitude spectrum of the signals presented in figure (c). (h) Amplitude spectrum of the signals presented in figure (d).

The first test (for both bandwidths) was to inject a weak sinusoidal single tone (1 *μ*V peak, 15 Hz) into tyrode solution and examine the recording capabilities of the Chebyshev notch channel in and without the presence of bipolar stimulation (140 Hz, 3 V peak, 100 *μ*s). Given 0.5–140 Hz bandwidth, the Chebyshev notch channel was able to record the weak sinusoidal single tone without (figures 9(a) and (e)) and in (figures 9(b) and (f)) the presence of bipolar stimulation. Finally, when the bandwidth was set from 0.5 to 250 Hz, the Chebyshev notch channel was again able to record the weak sinusoidal single tone without (figures 9(c) and (g)) and in (figures 9(d) and (h)) the presence of bipolar stimulation.

Having ensured that the designed Chebyshev notch channel was able to record a weak sinusoidal single tone during stimulation without facing saturation issues, the next step was to compare its artefact reduction capabilities with the capabilities of the Bessel notch channel. In these tests, ‘played back’ LFP signals (repetitions of an LFP segment lasting for 10 s, obtained from (Oostenveld *et al* 2011)) with two visible spectral peaks at approximately 167 Hz and 221 Hz were injected in tyrode solution from a waveform generator, as described in section 2.2 (figure 3). The goal of these experiments was to test all possible circumstances (in/without the presence of bipolar/unipolar stimulation) and prove that 1) the designed Chebyshev AFE can indeed provide a bandwidth that extends beyond the stimulation frequency of 140 Hz and 2) the application of the Chebyshev notch filter does not prevent the successful recording of frequencies that are close to the stop band (for instance the 167 Hz spectral peak of the LFP signal used in this series of experiments).

**Figure 10:**
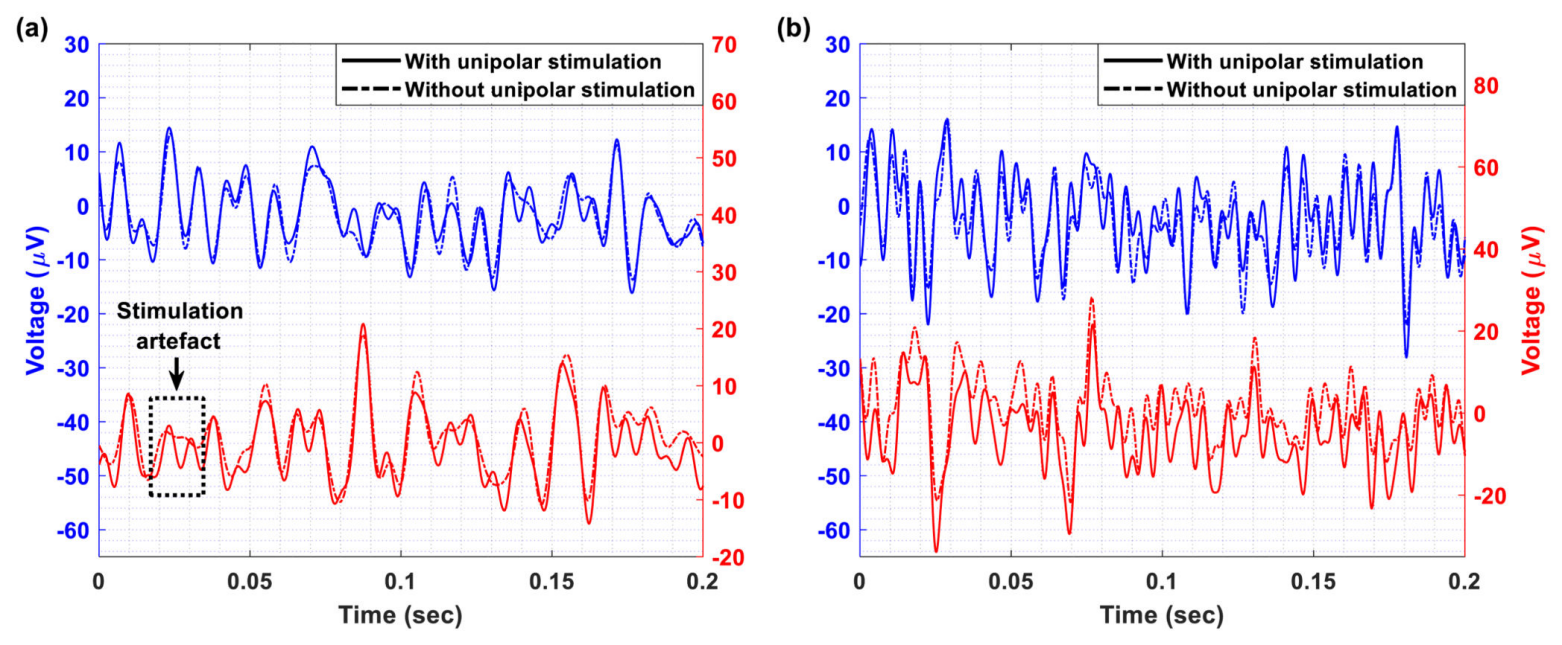
Detailed view of the time-domain LFP recordings taken from the Chebyshev (blue line corresponding to the left *y*-axis) and the Bessel (red line corresponding to the right *y*-axis) notch channels, with (solid line) and without (dash-dot line) unipolar stimulation. (a) The passband of both channels is between 0.5 Hz and 140 Hz. (b) The passband of both channels is between 0.5 Hz and 250 Hz.

**Figure 11:**
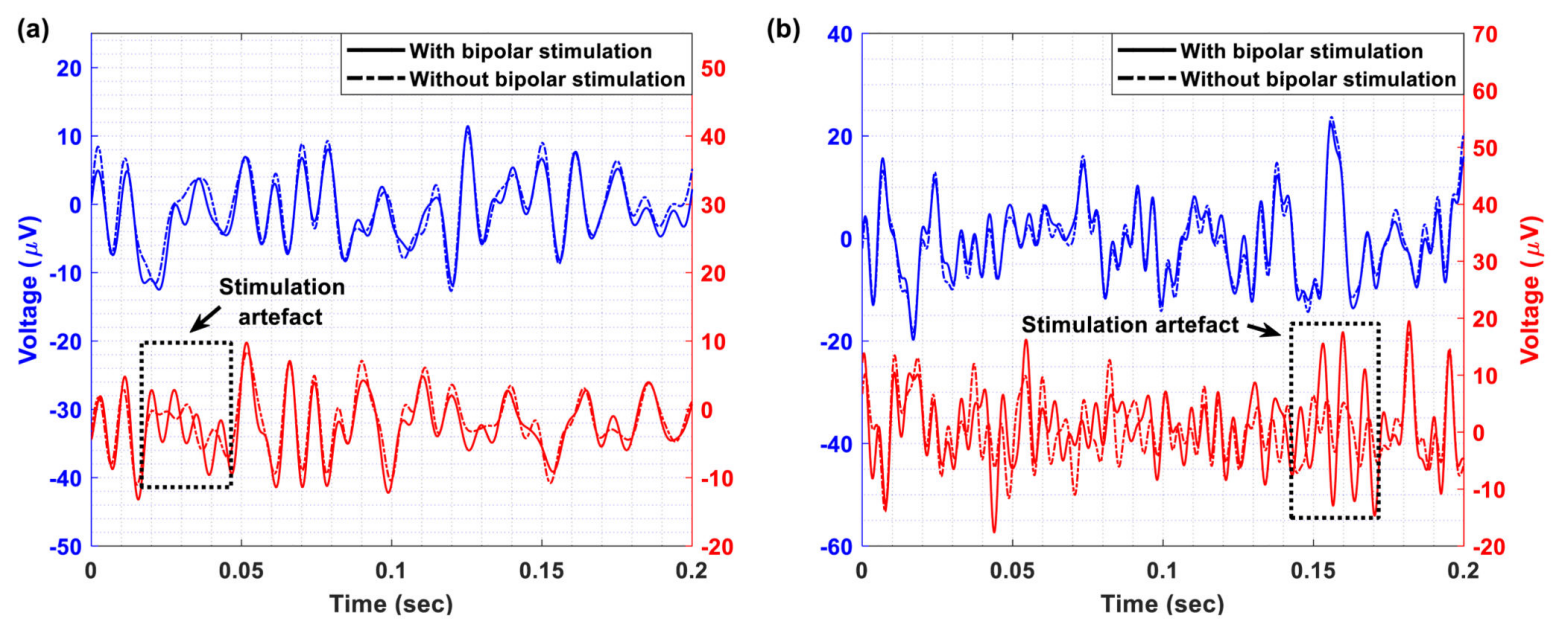
Detailed view of the time-domain LFP recordings taken from the Chebyshev (blue line corresponding to the left *y*-axis) and the Bessel (red line corresponding to the right *y*-axis) notch channels, with (solid line) and without (dash-dot line) bipolar stimulation. (a) The passband of both channels is between 0.5 Hz and 140 Hz. (b) The passband of both channels is between 0.5 Hz and 250 Hz.

Figures 10 and 11 illustrate detailed views of the time-domain LFP recordings taken from the Chebyshev (blue line) and the Bessel (red line) notch channels, with (solid line) and without (dash-dot line) unipolar (figure 10) and bipolar (figure 11) stimulation. In both figures, some stimulation artefacts appear in the LFP recordings collected by means of the Bessel notch channel. This observation leads to the conclusion that the Chebyshev notch channel provides more stable and reliable recordings of the LFP signals during stimulation in comparison to the Bessel notch channel. This is mainly attributed to the fact that the Chebyshev notch filter provides a stronger attenuation at the notch frequency than the Bessel notch filter (figure 5(a)). Moreover, a close inspection of the signals recorded by the Chebyshev notch channel in and without the presence of stimulation (figures 10 and 11) shows that the quality of the recovered LFP signals is not affected by ringing that could be introduced by the notch filter. This may be attributed to the fact that the ringing oscillations introduced by the Chebyshev notch filter as a response to DBS are low in amplitude and short in duration (bear in mind figure 4(d) where DBS pulses of the same duration were presented to the input of the high-order notch channel).

**Figure 12:**
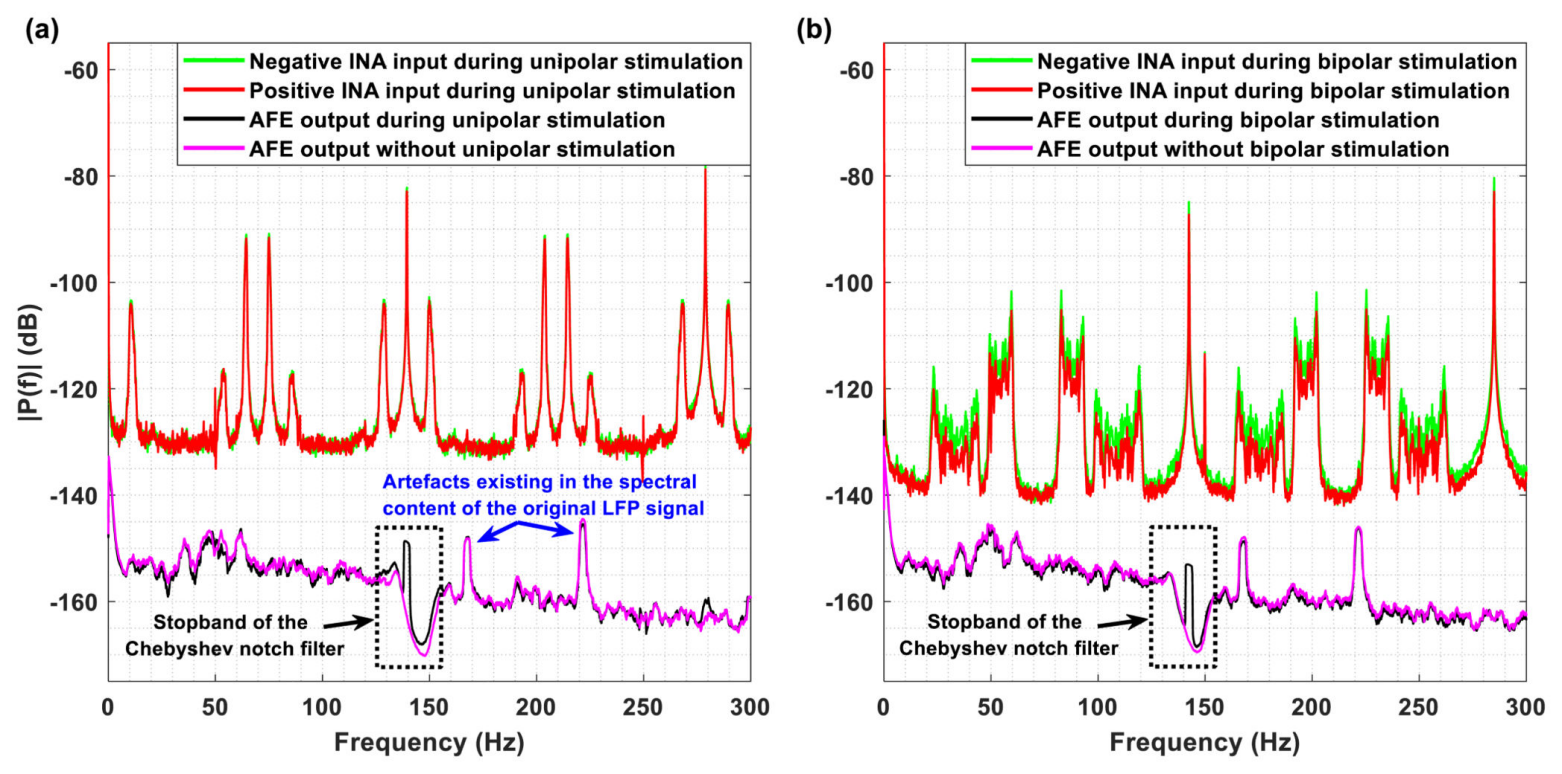
Amplitude spectrum (recorded from the Chebyshev notch channel) of the (1) signals entering the negative (green) and positive (red) inputs of the front-end INA during stimulation, (2) the AFE output voltage during stimulation (black), and (3) the AFE output voltage without the presence of stimulation (pink). (a) Unipolar stimulation setting, and (b) bipolar stimulation setting.

Furthermore, a graphical representation of the amplitude spectrum of the contaminating signals entering the positive (red colour) and negative (green) input of the front-end INA of the designed Chebyshev notch channel, along with the amplitude spectrum of the channel’s output in (black) and without (pink) the presence of stimulation, after digitally removing the 280 Hz harmonic from the recorded LFP signals during stimulation (spectrum in black), is depicted in figure 12. Figures 12(a) and (b) correspond to the unipolar and bipolar stimulation setting, respectively. It is clear that aliasing artefacts located at various frequencies that are not harmonic repetitions of the stimulation frequency (=140 Hz) exist in the spectrum of the contaminating signals. This finding is in accordance with results measured from existing DBS devices (Stanslaski *et al* 2012, Pinnell *et al* 2015). However, figure 12 shows that the amplitude spectrum of the channel’s output in the presence of either unipolar or bipolar stimulation is free from these artefacts and thus approximates the spectrum of the signals recorded without stimulation. This important finding could be attributed to the fact that the proposed AFE does not include passive filtering before the front-end INA. Front-end passive filters can lead to the degradation of the combined (passive filter plus INA) apparent CMRR of the front-end due to component mismatches (Casas *et al* 2009). The absence of such a passive filter network enhances the ability of the proposed AFE to reject common-mode disturbances stemming from the electrode-solution interface, thus offering a smooth spectrum at the output of the AFE and an artefact-free LFP recording in both unipolar and bipolar stimulation setups.

**Figure 13:**
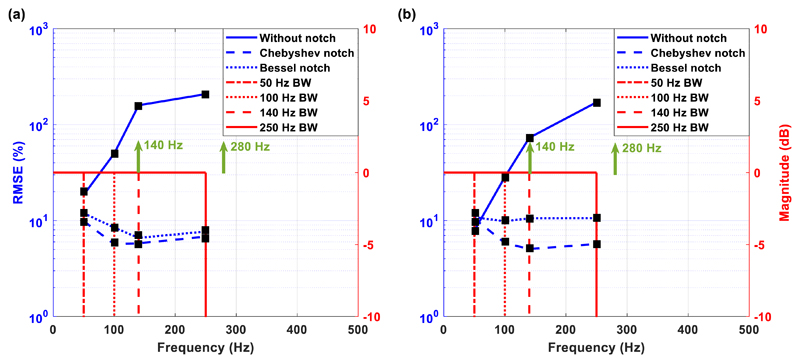
Normalised root mean square errors (RMSE) between the signals recorded in and without the presence of unipolar (a) and bipolar (b) stimulation. The green vectors show the main stimulation frequency component (=140 Hz) and the stimulation harmonic that is closer to the available passband (=280 Hz). The red lines (correspond to the right *y*-axis) show the available bandwidth (BW) for each recording trial and the blue lines (correspond to the left *y*-axis) depict the calculated RMSE values.

To quantify the differences between the recorded signals during stimulation and the ones recorded without stimulation, we used the normalised RMSE calculation. The normalisation for the RMSE calculation was performed over the range of the reference signal, which is the signal recorded without the presence of stimulation. Figure 13 shows the normalised RMSE values that represent the differences between the recorded signals in and without the presence of unipolar (figure 13(a)) and bipolar (figure 13(b)) stimulation for the Chebyshev (dashed blue line) and Bessel (dotted blue line) notch channels. The vertical red lines shown in the graph represent the amplitude Bode plots in each recording case. In other words, they describe the available passband, set by the application of a very steep real-time digital low-pass filter. To examine the benefits gained by the use of an analog notch filter for artefact suppression, we introduced a third AFE (solid blue line in figure 13), which does not include any analog notch filtering circuitry and suppresses the stimulation artefacts in the digital domain (by applying a very steep real-time digital low-pass filter). More specifically, this AFE includes a passive, 1st order low-pass filter at 8 kHz (to suppress high-frequency noise components), followed by the INA chip AD8420 from Analog Devices set to provide a gain of 20 dB.

Referring to figure 13(a), in the first bandwidth setting (0.5–50 Hz), the Chebyshev and Bessel notch channels present similar RMSE values, whereas the -channel without notch filter’ already shows a bigger error. In the next three bandwidth settings (100 Hz, 140 Hz and 250 Hz), the Chebyshev notch channel presents the lowest error, whereas the ‘channel without notch filter’ shows unacceptably high errors, which is attributed to the fact that: (a) aliasing artefacts exist in the frequency range 50–100 Hz, which is a finding that is in agreement with the literature (Pinnell *et al* 2015), and (b) the stimulation frequency (=140 Hz) is in the passband (when the digital low-pass filter is set at 140 or 250 Hz). The same conclusions are drawn from figure 13(b), where the Chebyshev notch channel presents the lowest error. As in the case of unipolar stimulation, the ‘channel without notch filter’ is characterised by unacceptably high errors at bandwidths greater than 50 Hz.

Another important observation is that the RMSE errors produced by the Chebyshev and Bessel notch channels decrease when the bandwidth increases from 50 Hz to 140 Hz and then slightly increase when the bandwidth is set at 250 Hz. This is attributed to a small intrinsic error that mostly comes from the dc offset voltage which is generated by the electrodes and is not completely rejected by the system. Hence, this small error in voltage is more apparent in smaller bandwidths where the recorded LFP signals are weaker due to filtering (0.5–50 Hz), decreases when the available bandwidth (and thus recorded LFP signal strength) increases (0.5–140 Hz) and, finally, slightly increases when the available bandwidth increases even more (0.5–250 Hz) since more interference leaks into the wide passband (the 280 Hz stimulation harmonic is getting closer to the passband).

### 3.4 Evaluation of the artefact suppression capabilities of the AFE by *in vivo* DBS tests

**Figure 14:**
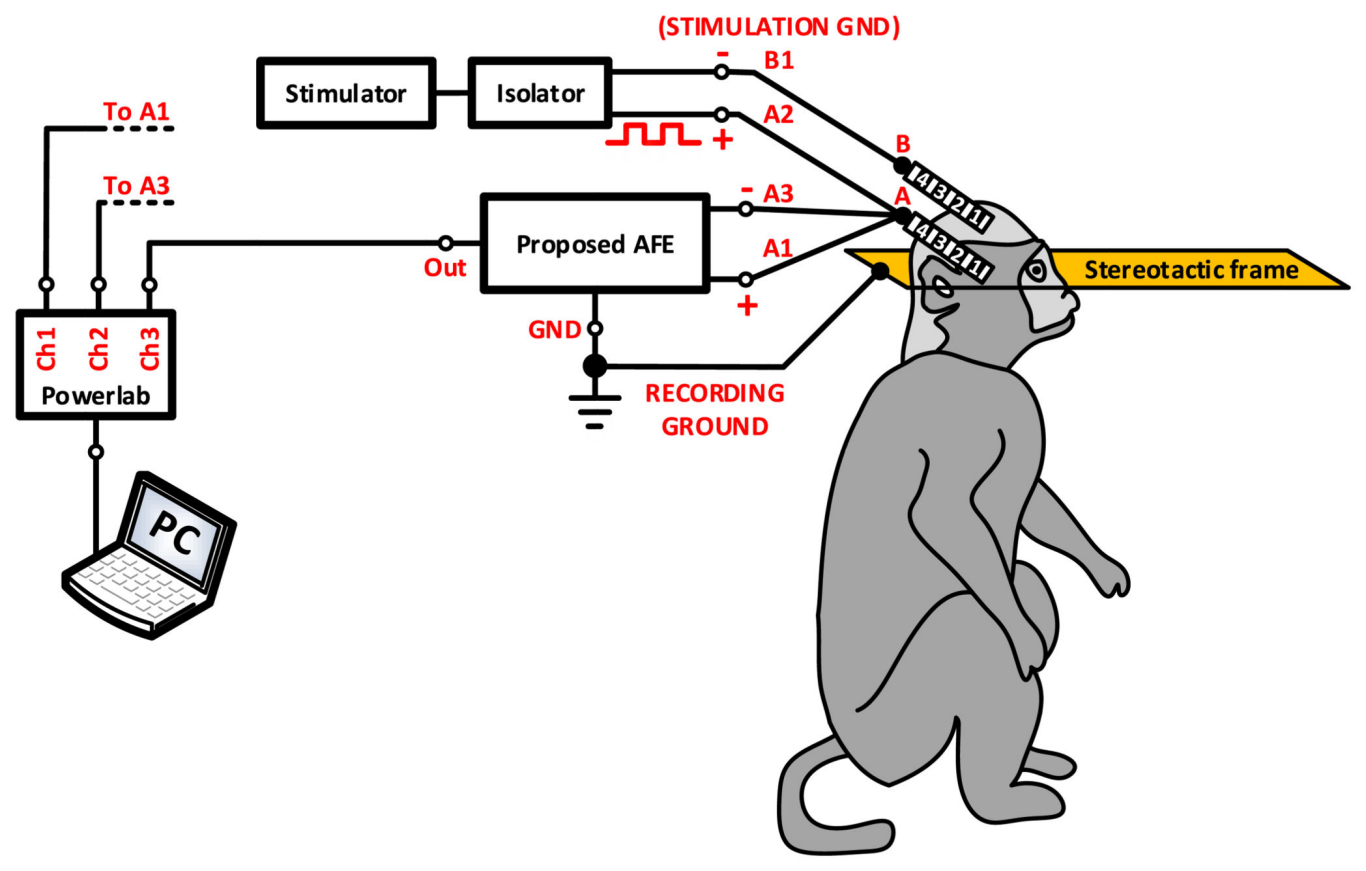
Experimental setup for evaluating the artefact suppression capabilities of the proposed Chebyshev AFE channel architecture *in vivo*. A DBS electrode (electrode A, model DB-2201, Boston Scientific Neuromodulation) was implanted into the thalamus of an anaesthetised non-human primate. The monophasic stimulation pulses (6 V peak-to-peak amplitude, 142 Hz frequency and 100 *μ*s pulse width) were delivered by means of a commercial stimulator (Grass, Astromed, Inc., USA). Unipolar stimulation was applied to contact A2 and LFP signals were differentially recorded through contacts A1 and A3. The stimulation ground was introduced into the brain tissue through a second electrode (contact B1, model 401261, St. Jude Medical) that was placed over the frontal cortex. The stimulation ground was electrically isolated from the mains using a commercial isolator (SIU5 stimulus isolation point, Grass, Astromed, Inc., USA).The non-human primate was under anaesthesia with the head held in a primate stereotactic frame, which was connected to the ground of our recording system. The LFP signals recorded by the proposed AFE were digitized at a sampling frequency of 20 kSPS (samples per second) and depicted on a computer by the Powerlab data acquisition system (ADInstruments).

To provide a proof-of-function *in vivo*, we recorded LFPs from the thalamus of a non-human primate, at the end of a non-recovery procedure that was performed for the primary purpose of another ongoing study. The experiments were approved by the local ethics committee at Newcastle University and performed under appropriate UK Home Office licenses in accordance with the Animals (Scientific Procedures) Act 1986. A female rhesus macaque was anesthetised with a ketamine/midazolam/alfentanil infusion and a segmented DBS electrode (electrode A, model DB-2201, Boston Scientific Neuromodulation) was implanted into the thalamus as shown in figure 14. The monophasic stimulation pulses (6 V peak-to-peak amplitude, 142 Hz frequency and 100 *μ*s pulse width) were delivered by means of a commercial stimulator (Grass, Astromed, Inc., USA). Unipolar stimulation was applied to contact 2 of electrode A (illustrated as A2 in figure 14) and LFP signals were differentially recorded through contacts 1 and 3 of electrode A (illustrated as A1 and A3 in figure 14, respectively). The stimulation ground was introduced into the neural tissue through contact 1 (illustrated as B1 in figure 14) of electrode B (model 401261, St. Jude Medical), which was placed over the frontal cortex. A commercial isolator (SIU5 stimulus isolation point, Grass, Astromed, Inc., USA) was used to electrically isolate the stimulation ground from the mains. The non-human primate was under anaesthesia during the entire experiment with the head held in a primate stereotactic frame, which was connected to the ground of our recording system. The LFP signals recorded by the proposed AFE were digitized at a sampling frequency of 20 kSPS and depicted on a computer by the Powerlab data acquisition system (ADInstruments).

**Figure 15:**
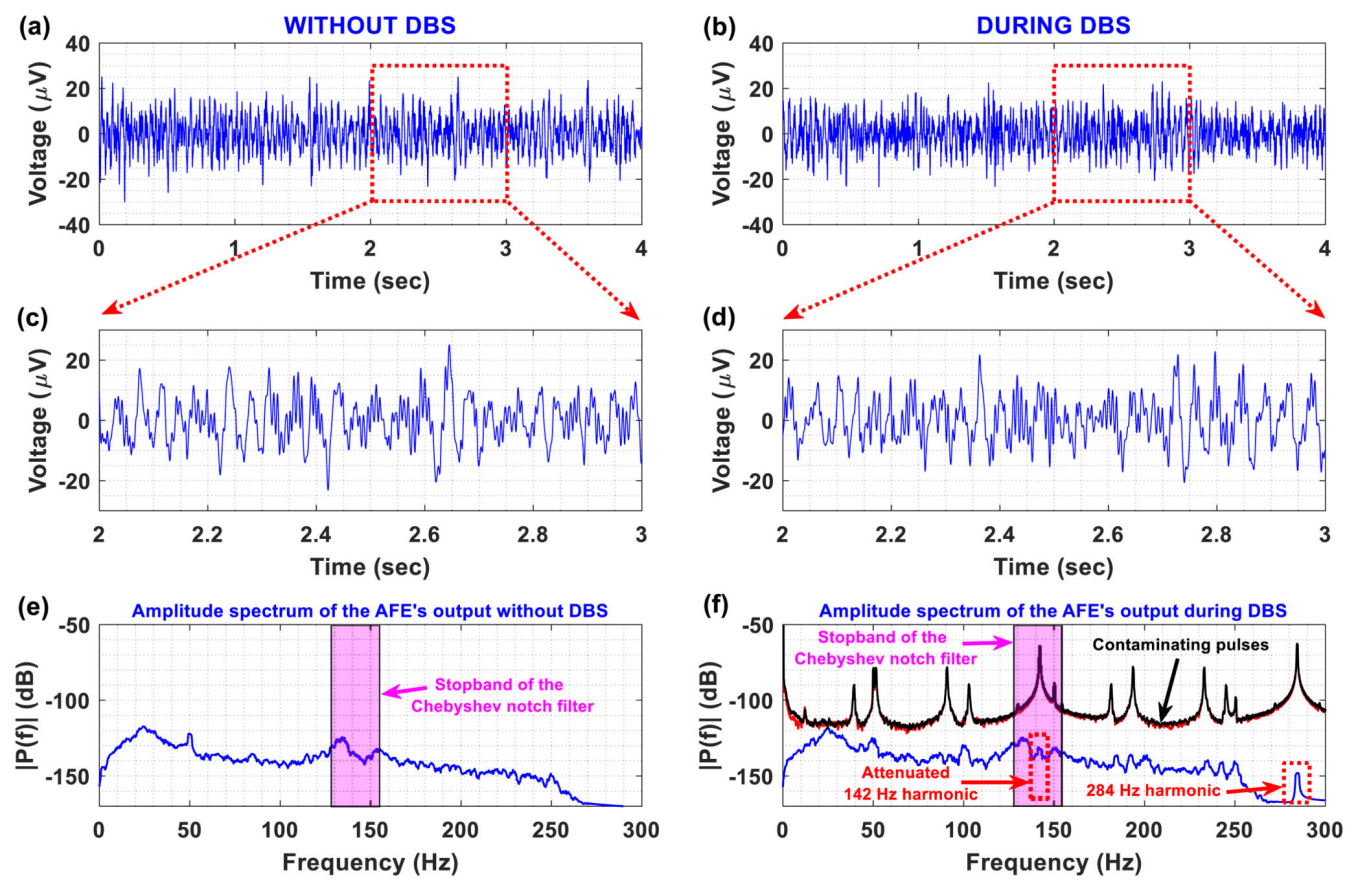
The proposed Chebyshev AFE architecture for artefact-free LFP recordings during unipolar DBS *in vivo*. LFP signals were recorded from the thalamus of an anaesthetised non-human primate in and without the presence of DBS with the experimental setup illustrated in figure 14. (a) Bipolar (differential) LFP recordings without DBS. (b) Bipolar (differential) LFP recordings during DBS. (c) Detailed view of the LFP recordings acquired without DBS. (d) Detailed view of the LFP recordings acquired during DBS. (e) Amplitude spectrum of the LFP signal recorded without DBS. (f) Amplitude spectrum of: (1) the LFP signal recorded during DBS (blue line), and (2) the stimulation pulses presented at the positive (red line) and negative (black line) inputs of the front-end instrumentation amplifier. It is clear that the proposed artefact suppression strategy (analog notch filtering at 140 Hz and digital low-pass filtering at 250 Hz) allows for artefact-free LFP recordings during DBS (observe the 142 Hz stimulation fundamental frequency, which has been strongly attenuated by the high-order notch filtering action).

As shown in figures 15(a) and (b), the Chebyshev notch channel can provide artefact-free LFP recordings during DBS. Moreover, after examining the detailed views of the LFP recordings acquired without and in the presence of DBS (figures 15(c)–(f)), we conclude that: (1) the stimulation artefacts (at 142 Hz and 284 Hz) induced by DBS have been significantly suppressed (blue line in figure 15(f)), (2) the amplitude spectrum of the LFP signals recorded during DBS (figure 15(f)) is free from aliasing artefacts, which is in full agreement with the *in vitro* experimental results shown in figure 12, and (3) the contaminating 142 Hz DBS pulses are successfully suppressed by 68 dBs (amplitude spectrums in red and black in figure 15(f)) thanks to the combined notch filtering action and the front-end INA’s (high) CMRR.

## 4 Discussion

### 4.1 Methodological significance

The analog filtering strategy used in the proposed AFE architecture is an effective approach to adequately suppress stimulation artefacts. In our application, the stimulation artefact and the signal of interest are highly overlapping both in the time and frequency domain. More specifically, the stimulation frequency (=140 Hz) and its first two harmonics (280 and 420 Hz) are located within the desired system bandwidth (0.5–500 Hz). Therefore, the stimulation artefact and its harmonics could not be separated from the neural signals of interest using an analog high-order low-pass filter, which was the strategy employed by Rossi *et al* (2007).

An alternative approach that has been extensively adopted is to provide a switching circuit that disconnects the front-end leads of the amplifier during stimulation (Rossi *et al* 2007). This strategy is effective for applications, such as transcranial magnetic stimulation (Paus *et al* 2001, Fuggetta *et al* 2005, Van Der Werf and Paus 2006, Van Der Werf *et al* 2006) and evoked potentials (Knaflitz *et al* 1988), because in these setups the stimulation artefact and the signal of interest are well separated in the time domain but highly overlapping in the frequency domain (Rossi *et al* 2007). Since our aim was to provide neural recordings during stimulation, we avoided employing this technique.

Moreover, in a typical stimulation therapy (for PD or Dystonia) the neural signals of interest, which typically are on the order of 1–10 *μ*V when measured from DBS electrodes, are up to six orders of magnitude weaker than the stimulation pulses. As a result, a high gain is required to make the neural signals detectable by the front-end electronics and the subsequent ADC blocks. However, this high amplification is applied on both neural signals and stimulation artefact, which often leads to the saturation of the front-end amplifier. Hence, the approach to completely shift the stimulation suppression to the digital domain by using FIR filtering or template subtraction techniques increases the risk of saturation (Rossi *et al* 2007).

Besides that, template subtraction techniques suffer from varying artefact morphology stemming from undersampling the artefact shape and misalignment between stimulation and sample timing (Qian *et al* 2017, Zhou *et al* 2018). Similarly, adaptive filtering methods filter the stimulation pulse (Mendrela *et al* 2016) or the artefact recorded on a neighbouring channel (Basir-Kazeruni *et al* 2017) in order to estimate and subtract the artefact while filter coefficients are adapted. According to Zhou *et al* (2018), these subtraction methods can be implemented with low latency, but require artefact detection, template building and on-board memory for template storage. To avoid signal distortion, templates must be regularly updated to track any changes in artefact shape or stimulation waveform. Furthermore, estimated templates often take time to converge, resulting in varying levels of cancellation over time. Finally, high dynamic range front-ends are required for subtraction and component decomposition techniques, since the undistorted artefact waveform has to be recorded.

Reconstruction methods remove samples contaminated with artefacts and replace them with interpolated values. More specifically, sample-and-hold methods hold over the last known good sample for the duration of each artefact (Montgomery *et al* 2005, Hartmann *et al* 2015). This procedure requires only a single sample of memory, but may cause significant distortion (Zhou *et al* 2018). To reduce distortion, samples may be replaced by linear interpolation between the nearest clean samples (Heffer and Fallon 2008, Zhou *et al* 2019), an estimation from a learned Gaussian probability density (Hoffmann *et al* 2011) for data segments, or a reconstruction using cubic spline interpolation (Waddell *et al* 2009). Although simple to implement, reconstruction methods require artefact detection. This can be done using blind detection algorithms (Montgomery *et al* 2005, Heffer and Fallon 2008, Hoffmann *et al* 2011), or using timing indicators from the stimulator (Zhou *et al* 2019). These methods lose information during the artefact, degrading the achieved SNR (Zhou *et al* 2018).

Taking into consideration the above discussion of the digital artefact suppression approaches proposed so far, it can be argued that an alternative strategy for recording in real time artefact-free LFP signals during stimulation could be based on the efficient application of a combination of analog and digital filters. The preservation of real-time operation is particularly important in neuromodulation because the stimulation must change in real time based on the measured state of the neural network (Stanslaski *et al* 2012). The strategy of removing all of the artefacts by employing (usually high-order) digital filtering techniques could lead to significant delays in data processing and challenge the practicality of a real-time, closed-loop system that would employ a digital only artefact removal strategy; thus this strategy was also put aside.

The finally adopted approach was to introduce a Bainter analog notch filter to increase the available bandwidth by suppressing the artefact originating from the stimulation frequency (=140 Hz) and apply high-order low-pass filtering to reject the higher-frequency harmonics. For convenience and testing purposes the low-pass filtering was realised in the digital domain to facilitate the experimental study of our approach. Conceivably, however, a high-order analog low-pass filter could also be used to reject high-order harmonics albeit at the expense of size for the externalised device and limited flexibility during the experimental testing of our approach. The Bainter notch filter topology was selected because its Q is dependent on the gain of the amplifiers as opposed to component matching. Consequently, the notch depth is not sensitive to temperature drift or aging (Baker 2015). The analog notch filter introduces a negligible delay in the signal processing chain. The digital low-pass filtering block was provided by the Powerlab 16/35 system and introduced a processing delay of 75 ms. This delay is in full agreement with the delays introduced by wearable recording systems that apply real-time digital signal processing (Salehizadeh *et al* 2016).

Another reason for introducing an analog notch filter in the signal chain is to suppress the stimulation interference produced by the electrode/tissue impedance mismatch. This mismatch exists even in a symmetric sensing and stimulation setup and is hard to be controlled within a biological environment (Stanslaski *et al* 2012). Therefore, an AFE that includes an eight-pole Bainter notch filter with Chebyshev response was designed, developed and tested *in vitro* and *in vivo.* Besides that, a comparison, in terms of recording quality and artefact suppression capability, between the designed AFE, an identical AFE employing an 8th order Bessel notch filter, and an AFE that does not include any analog band-stop filtering and rejects all the artefacts digitally, was drawn and measured results were presented.

Since the next harmonic after the main stimulation frequency is at 280 Hz, we decided to gradually increase the available bandwidth from 140 Hz to 250 Hz and calculate the normalised RMSE between the recorded signals in and without the presence of stimulation. In both unipolar and bipolar stimulation, when the bandwidth is restricted between 0.5 and 50 Hz, the RMSE values of the three channels are kept low. However, the channel lacking notch filtering presents unacceptably high RMSE values when the bandwidth is extended beyond 50 Hz. This finding, which stresses the necessity of using an analog notch filter for artefact suppression, is in accordance with results measured from existing DBS systems that offer a passband reaching 100 Hz. Those results showed that prominent stimulation artefacts existed on the raw LFP trace, consisting of both harmonic repetitions of the stimulation frequency and aliasing artefacts (Pinnell *et al* 2015). Regarding the Chebyshev and Bessel notch channels, no significant change in the calculated RMSE was introduced by the extension of the recording bandwidth to 250 Hz, which leads to the conclusion that the artefacts introduced by the 280 Hz harmonic (and higher ones) do not significantly affect the quality of the recorded signals when adequately suppressed by a high-order low-pass filter at 250 Hz.

A thorough examination of the measured results presented in this paper leads to the conclusion that the sensing and stimulation artefact suppression capabilities of the Chebyshev notch channel outperform the capabilities of the Bessel notch channel. Another important finding is that the proposed AFE architecture provides LFP recordings that are not affected by aliasing artefacts located at frequencies that are not harmonic repetitions of the stimulation frequency. Hence, bearing in mind the desire for wide bandwidth LFP recording with DBS artefacts suppressed, the approach studied here suggests that cascading two analog high-order notch filters at 140 and 280 Hz with a steep, high-order, also analog, low-pass filter of a cut-off frequency ~400 Hz should enable practical, low delay, high-quality and higher bandwidth (~400 Hz) LFP recording with DBS artefacts strongly suppressed; at the expense of somewhat increased size and power consumption of the externalised AFE. The full digital realisation in the Lab (e.g. via Powerlab) of the same architecture (two notch filters and a low-pass one) introduces a total approximate delay higher than 0.6 s [2 × 270 ms (notch filters) + 75 ms (low-pass)] which challenges the practicality of a fast, closed-loop neuromodulation system.

One of the important merits of the proposed approach is that biosignal blanking during stimulation is avoided. Moreover, the proposed artefact suppression strategy allows for artefact-free LFP recordings during monophasic DBS, which can be perceived as the worst case scenario, since in biphasic DBS the artefact and the electrochemical DC offsets (Zhou *et al* 2018) produced are weaker (figures 4(d) and (e)). Indeed, it should be stressed that in both *in vitro* and *in vivo* experimental setups, LFP signals were successfully recorded during stimulation *even in the presence of inherent asymmetries* introduced by the use of segmented DBS electrodes that allowed differential-mode interference to enter the signal chain. Furthermore, the design decision to directly connect the front-end amplifier to the DBS electrodes allowed us to avoid the introduction of a passive high-pass filter network at the first stage of the AFE and the subsequent noise deterioration and CMRR reduction this strategy would entail. The input bias current of the front-end INA (150 nA maximum) is lower than the limit imposed by the IEC 60601-1 standard (maximum allowable patient auxiliary current for Type B, normally connected applied parts equals 10 *μ*A). Thus, the designed AFE complies with safety requirements.

Finally, when pairs of electrodes are used for measuring differential voltages from the human body (invasively or non-invasively), it is recommended to use the same material for each of the electrodes because, in such a case, their half-cell potentials are approximately equal. According to Webster (2010), this strategy: (1) ensures that the net DC potential seen at the input of the amplifier connected to the electrodes is relatively small, and (2) minimizes possible saturation effects in the case of high-gain direct-coupled amplifiers. Hence, since our proposed high-gain front-end INA (with high-pass characteristics) differentially records LFP signals from two same material (platinum-iridium) contacts of a DBS electrode, the differential DC offset that is produced at the electrode-tissue interface is in the order of tens of millivolts (Denison *et al* 2007), and it is thus removed by our system (the maximum dc offset rejection that can be accomplished by our system in its current format is ±32 mV, which is in full agreement with the rejection offered by recent bidirectional neural interface systems (Greenwald *et al* 2016)), as verified by the *in vitro* and *in vivo* measured experimental results presented here. Based on the datasheet of the front-end INA (AD8429) chip, the maximum electrochemical DC offset that can be rejected by our system is approximately equal to 2.4 V, so long as it is presented as a common-mode signal at both inputs of our Chebyshev notch AFE.

### 4.2 Limitations and further improvements

All of the practical merits described above, are achieved at the expense of signal loss in the frequency band ranging from 125 to 155 Hz and the appearance of noise in this frequency band when extremely weak (0.32 *μ*V rms) LFP signals, which are close to the Chebyshev notch channel’s noise floor (~0.1 *μ*V rms), are recorded (figure 8(b)). Crucially, the impulse response of the analog notch filter when stimulated by a DBS pulse is characterized by low-amplitude and short-duration transient ringing (figures 4(d) and (e)), which, as the measured results in figures 8(c), 10 and 11 show, does not affect the quality of the recorded LFP signals. However, even this short and weak ringing can be avoided by adding an analog multiplexer which would allow the user to introduce, through software, the analog notch filter in the signal chain just before the onset of DBS and exclude it from the signal chain before the termination of stimulation, e.g. 15 ms before the last stimulation pulse. In this way, the weak ringing effect that may be introduced by the Chebyshev notch filter after the last DBS pulse it senses, would not appear after the (actual) last stimulation pulse, and thus it would not interfere with evoked resonant neural activity, which is a physiological signal of interest that appears 4 ms after the (actual) last DBS pulse and lasts for ~20 ms (Sinclair *et al* 2018). Furthermore, in a future version of this AFE (comprised, for example, of two high-order analog notch filters and one high-order analog low-pass filter, as explained above) the introduction of digitally selectable/tunable notch and cut-off frequencies would provide the researchers and clinicians with the ability to suppress more than one stimulation rate and preserve wide bandwidth recording.

Finally, the existence of small intrinsic errors produced by the Chebyshev and Bessel notch channels (figure 13) mainly results from the dc offset voltage, which is produced by the electrodes and is not completely rejected by the system. However, further rejection of this differential dc offset (up to 85 mV of differential dc offset voltage rejection can be achieved, as reported in table 2) could be accomplished by introducing a passive 1st order high-pass filter between the front-end INA and notch filtering stages (in other words between stages 1 and 2 of the current AFE design shown in figure 1). The addition of this high-pass filter block would introduce a small delay in the transient response of the system (Henderson and Kautz 1958), however, this delay may be considered tolerable taking into account the enhancement this strategy could offer in the recording capabilities of the Chebyshev notch channel during stimulation.

### 4.3 Pathophysiological significance

The key aim for this system is to remove existing constraints for clinical neuroscience discovery with bi-directional neural interfaces. The major attributes of our system are its wide pass band and low noise floor relative to other devices allowing the recording of signals during stimulation at the same site with greater resolution, especially at higher frequencies. An alternative approach is to shift attention from LFPs in subcortical nuclei to electrocorticographic recordings as tractable feedback biomarkers for closed-loop stimulation (De Hemptinne *et al* 2015, Herron *et al* 2016, Swann *et al* 2016, 2018). In some cases, this is necessary as electrocorticographic signals have better signal-to-noise ratios and are spatially separated from DBS sites, so they are less corrupted by stimulation-induced artefact.

However, subcortical recordings have their own merits. These include the inherent convergence in basal ganglia targets, so that extensive cortical regions can be modulated, and clearer pathological correlates (Oswal *et al* 2013). Moreover, the recording of signals from the same electrodes as used for DBS limits instrumentation of the brain and thereby the incremental morbidity and expense. The currently described system facilitates consideration of LFP activity as a source of feedback control. Specifically, it widens the potential feature space beyond the beta band (Little *et al* 2013, Priori *et al* 2013, Arlotti *et al* 2018) to lower amplitude, higher frequency activities. These include finely tuned gamma activity centred around 70 Hz and associated with dyskinesias (Fogelson *et al* 2005), and high frequency oscillations of over 200 Hz in frequency, together with related phase amplitude coupling which have both been linked to bradykinesia and rigidity (Meidahl *et al* 2019). In addition, stimulation evoked subcortical potentials have high frequency components that may carry information about targeting and motor impairment in Parkinson’s disease (Sinclair *et al* 2018). A richer feature space also improves the capability of machine learning approaches to identify control signals (Shah *et al* 2018, Yao *et al* 2018). In its current embodiment, the large power dissipation would not allow for placement of the circuit in an implantable system. The intention is to achieve a more complete sampling of the physiomarker space, and based on what is discovered, define a bespoke application specific integrated circuit (ASIC) that would provide the resolution required within the power constraints of the implant.

To sum up, from the pathophysiologic point of view, being able to extend the available bandwidth for LFP recording from the target stimulation site constitutes pivotal progress for developing novel closed-loop neurostimulation systems that use low- and higher-frequency LFPs as control signals. The *in vivo* experimental results presented in this study show that our recording system: 1) does not saturate during DBS, and 2) is able to provide artefact-free LFP recordings during DBS. The next step towards developing the aforementioned closed-loop DBS technology would be to use the proposed AFE architecture in a clinical study to continuously record LFPs during DBS from the STN of parkinsonian patients and, more specifically, from the neural tissue surrounding the stimulating electrode. This study would allow the identification of changes in LFP rhythms induced by DBS and the determination of features extracted from the LFP signals that could be used to regulate and optimise ongoing DBS.

## 5 Conclusion

The novel and versatile Chebyshev notch channel designed, realised and tested *in vitro* and *in vivo*, allows for real-time, low-noise and artefact-free LFP recordings during DBS for a bandwidth of 0.5–250 Hz. Proof of the proposed channel’s recording and artefact suppression capabilities has been provided and its performance has been evaluated quantitatively by means of a series of *in vitro* and *in vivo* experiments and comparisons with commercial high-performance biopotential acquisition systems. It has been proven that the designed AFE is able to reliably record weak LFP signals (1–10 *μ*V peak in figures 10, 11 and 15), in and without the presence of either unipolar or bipolar DBS, which renders it a functional and practical AFE architecture to be utilised in a wide range of applications and environments. This work is the first step towards developing a closed-loop neurostimulation system that uses low and higher-frequency LFPs as control signals.

## Figures and Tables

**Table 1:** Key AFE requirements for reliable acquisition of LFPs during deep brain stimulation (DBS).

Property	Value	Units/comments
Gain	⩾60	dB
Noise power spectral density estimate	⩽100	$\text{nV}{\left(\sqrt{\text{Hz}}\right)}^{-1}$
Integrated noise	⩽100	nV rms (0.5–500 Hz)
CMRR	⩾100	dB (DC to 60 Hz)
Differential DC offset to tolerate	Tens of	mV
Hours of continuous operation	⩾24	h
Input dynamic range	⩾ ±200	*μ*V
High-pass corner	0.5	Hz
Low-pass corner	500	Hz

**Table 2:** Key properties of the Chebyshev notch AFE.

Property	Value	Units/comments
Supply voltage	±5, ±2.5	Volts
Gain	60, 80	dB (programmable)
Integrated noise	26	nV rms (0.5–40 Hz)
	33	nV rms (0.5–100 Hz)
	96	nV rms (0.5–500 Hz)
CMRR	130	dB (DC to 60 Hz)
Maximum tolerable differential DC offset	±32/85^a^	mV
Dynamic range	±2.3	mV (peak), gain = 1000
	±230	μV (peak), gain = 10000
SNR	30	dB (minimum)
Nonlinearity	<0.2%	THD
High-pass corner	0.5	Hz
Low-pass corner	500	Hz
Total current consumption	32	mA
Hours of continuous operation	28	h (900 mAh battery)

a Measured differential DC offset rejection of 85 mV is achieved when a 1st order analog 0.5 Hz high-pass filter is cascaded after the front-end INA (stage 1 of the current AFE).
